# Tropical climate modes control strength and distribution of thermal stress mitigation in a coral reef refugia

**DOI:** 10.1038/s41598-026-52941-6

**Published:** 2026-06-21

**Authors:** Hana Camelia, Thomas Felis, Jessica A. Hargreaves, Martin Kölling, Sander Scheffers, Suchana Chavanich, Chalermrat Sangmanee, Marlene Wall

**Affiliations:** 1 MARUM – Center for Marine Environmental Sciences, University of Bremen, 28359 Bremen, Germany; 2 Oceans Institute, The University of Western Australia, Perth, 6009 Australia; 3 Department of Marine Science, Faculty of Science, Chulalongkorn University, Bangkok, 10330 Thailand; 4 Department of Marine and Coastal Resources, Phuket Marine Biological Center, Phuket, 83000 Thailand; 5 GEOMAR Helmholtz Centre for Ocean Research Kiel, 24148 Kiel, Germany

**Keywords:** Climate sciences, Ecology, Ecology, Ocean sciences

## Abstract

Tropical coral reefs are threatened by rising seawater temperatures. However, cold-water intrusions to coral reefs by oceanic processes such as upwelling or internal waves, which propagate along the thermocline, can offer refugia from global warming. How reef temperatures and coral responses vary in such locations remains poorly understood due to limited in-situ observations. We present monthly subsurface temperature and coral response records based on coral skeletal geochemistry from the Andaman Sea, a known thermal refugia. Coral Sr/Ca-temperature indicates subsurface reef cooling varied at interannual to decadal timescales, driven by large-scale wind and thermocline changes, and was most pronounced during the combined positive Indian Ocean Dipole and El Niño event of 1997/98. Coral carbon isotopes indicate relative shifts from autotrophy to heterotrophy during most coral bleaching events observed in the Andaman Sea, except during the 1998 event when we found strong and widespread subsurface reef cooling by thermocline shoaling leading to enhanced influence of internal waves. Our results indicate tropical climate modes such as the Indian Ocean Dipole and the El Niño-Southern Oscillation controlled the strength and spatial distribution of thermal stress mitigation by oceanic processes through time, suggesting some coral reef refugia will be variable under future climate change.

**Supplementary Information:**

The online version contains supplementary material available at 10.1038/s41598-026-52941-6.

## Introduction

Climate change poses a major threat to tropical coral reef ecosystems by causing prolonged thermal stress during marine heatwaves, as a consequence of globally rising seawater temperatures^1^. Marine heatwaves often lead to mass coral bleaching, which is a breakdown of the symbiotic relationship between corals and their photosynthetic microalgae, resulting in the loss of algal symbionts and subsequent starvation^2^. If thermal stress becomes more severe or prolonged, coral mortality can occur^2^. In some cases, more temperature-sensitive coral species may die immediately following extreme heatwaves^3^. Marine heatwaves have become more frequent, intense, and longer in duration, resulting in recent global coral bleaching events^4,5^. These trends are projected to accelerate under future climate change further pushing coral reef ecosystems to the limits of their resilience^6^.

Not all coral reefs respond to marine heatwaves similarly as certain locations can provide refugia from rising temperatures. Upwelling-influenced reefs are subjected to mixing and advection of cold, nutrient-rich deep water^7^. These processes are caused by the upward movement of thermal/density layers in the water column (i.e., thermocline/pycnocline shoaling) or by internal waves^7^. Internal waves, also referred to as internal tides or large-amplitude internal waves (LAIW), propagate along density gradients and are induced by strong tidal flows interacting with bottom topography^7–11^. Thus, upwelling-influenced reefs experience reduced thermal stress^7,10–14^ and often increased food availability^11,15,16^. The latter can result in a higher thermal resistance during heat stress events^17–20^. For instance, localised upwelling in the eastern tropical Pacific^16^ and internal waves in the Andaman Sea^12,21^ mitigated the impacts of thermal stress during previous global coral bleaching events. Consequently, these reefs are considered to more likely survive under ongoing and future climate change^22^. These refuge sites have become a priority for coral reef conservation and restoration^23^ as they can also provide a major source of larval dispersal to replenish degraded reefs, as demonstrated in the Great Barrier Reef following repeated bleaching events^24^.

**Fig. 1:**
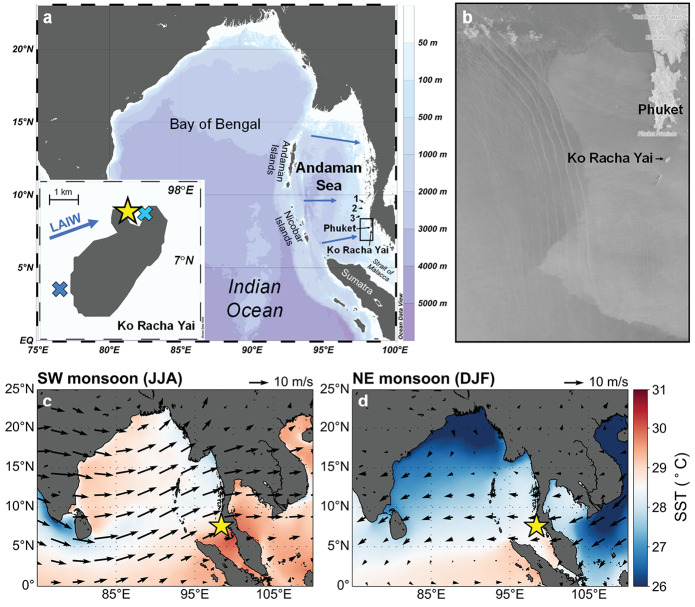
Map and climatology of the Andaman Sea and Bay of Bengal, northeastern Indian Ocean. (**a**) Bathymetry and large-amplitude internal waves (LAIW) propagation. Inset: coral site (yellow star) at Siam Bay, Ko Racha Yai (Thailand) and in-situ temperature sites (light blue cross^43^, dark blue cross^12,44^). Direction of LAIW propagation based on leading solitons of LAIW packets captured by satellite-based sensor images^45^. In-situ temperature sites at southern Andaman Sea islands: 1 Surin Island^12,46^, 2 Tachai Island^12^, 3 Miang Island^47,48^. Map generated with Ocean Data View^49^. Rectangle represents domain of (**b**) modified Copernicus Sentinel 1A synthetic aperture radar (SAR) image (April 2018) visualising LAIW packets moving toward Ko Racha Yai and Phuket. Figure processed in Copernicus Browser. Climatology (1985–2010) of sea surface temperature (SST) (NOAA CRW^25^) and winds (850 hPa level) (ERA5^50^) during (**c**) southwest (SW) (June-July-August, JJA) and (**d**) northeast (NE) (December-January-February, DJF) monsoon seasons.

Satellite sea surface temperature (SST) products are widely used in reef studies to monitor thermal stress associated with coral bleaching^3,25–27^, but they have several limitations. Current high-resolution satellite SST^25^ observations record only the upper few mm of the water column with 5 km spatial resolution and are unable to capture reef-scale temperature variability induced by deep-water shoaling below this thin surface layer^12,28–30^. The influence of cold-water intrusions into shallow reef habitats is ubiquitous in tropical ocean regions^7^. One upwelling feature, the occurrence and propagation of LAIW, can be captured by synthetic aperture radar and optical sensors^8^ (e.g., Fig. 1a,b) and provide a first indication of their potential for reef cooling. However, cold-water intrusions of LAIW, internal waves, or internal tides on the reefs rarely extend to the surface and often operate on small spatial scales (<1 km)^22^. Other limitations of satellite SST are temporal and spatial averaging^31,32^ that are blind to small-scale oceanographic features like internal waves. Some satellite SST products include only night-time observations^25,33^, which may result in an incomplete representation of the temporal variability of upwelling influence. In addition, different SST products can disagree in the extent they capture the conditions on the reefs during bleaching events^34^ and therefore may not capture how spatially variable protection from bleaching can be due to upwelling influence^12,14^. Recent efforts using ultra-high resolution (<2.5 km) global ocean models are still limited to single-year outputs^22^, while longer model simulations with lower resolution^9,35,36^ lack validation with rare in-situ observations of subsurface temperature. Consequently, we still lack a clear understanding of whether interannual to decadal changes in the strength of upwelling influence exist and how these affect the temporal and spatial variation of thermal stress mitigation at reef refuge sites.

Recent reef studies, however, have underscored the importance of temperature variability for reef health and persistence in a future changing ocean^10,37–39^. In particular, upwelling-influenced reefs were shown to experience reduced thermal stress during marine heatwaves, which mitigated coral bleaching responses, rendering healthier corals^12,14,21,40^. Furthermore, laboratory-based studies have been used to simulate the influence of LAIW-induced temperature variability during a marine heatwave on coral stress response^17,37^, providing proof-of-concept for their ability to abate heating. Corals’ ability to derive energy from heterotrophic feeding has also been shown to influence the extent of bleaching following damage to the phototrophic symbiont system. For example, heterotrophic feeding has been demonstrated to promote coral resilience during bleaching and recovery by providing a fixed carbon source to meet metabolic energy demand and to maintain energy reserves and tissue biomass^18–20^. Energy subsidies through heterotrophic feeding can be enhanced during marine heatwaves in upwelling-influenced reefs^41^. Enhanced nutrients caused by upwelling^11,15,16^ can increase phytoplankton concentrations and reduce photosynthetic active radiation, which has also been linked to bleaching mitigation^42^. Therefore, upwelling may provide protection during marine heatwaves, both by mitigating heat stress and by enhancing heterotrophic feeding. All these mechanisms (heat stress mitigation by upwelling or dynamic adjustment of trophic strategy) can help corals to persist in a future changing ocean, but we have little understanding of their spatial and temporal variation and link to the strength of upwelling influence. The lack of long-term, continuous reef monitoring and temperature observations at depths where corals grow limit our knowledge on how reef-scale temperatures and coral responses to thermal stress have been changing through time at refuge sites, which may play a key role in the protection of reefs in a future changing ocean.

Here we present monthly-resolved skeletal geochemical and isotopic records of a common Indo-Pacific shallow-water coral (*Porites lobata*) from a prominent reef refuge site^51^ in the southern Andaman Sea (Ko Racha Yai, Thailand), northeastern Indian Ocean (Fig. 1). The Andaman Sea is an ocean region exposed to extraordinarily pronounced LAIW^8^. The area is under the influence of the Asian monsoon, with its wet southwest (SW) monsoon during summer (May-October) and dry northeast (NE) monsoon during winter (November-April)^12^ (Fig. 1c,d). We use coral Sr/Ca, a widely applied temperature proxy in tropical corals^52–55^ for studying seasonal to interannual variability over the last centuries^56–^^59^ and during time intervals of the Holocene^60^, the last glacial and deglacial^61,62^, the last interglacial^63–65^, and the penultimate glacial^66^. This proxy allows us to reconstruct subsurface temperature variability at coral depth (~10 m), which is influenced by thermocline shoaling and LAIW. In parallel, coral stable carbon isotopes (δ^13^C) are assessed as they reflect the combined effects of various parameters including the availability of light for photosynthesis of the coral’s algal symbionts, the coral’s growth rate, and the contributions of autotrophy and heterotrophy to the coral’s diet^67–71^. In addition to these metabolic controls, coral δ^13^C is influenced by changes in the δ^13^C of dissolved inorganic carbon (DIC) of seawater (δ^13^C_DIC_), which can be affected by atmospheric changes on a global scale and river discharge and upwelling on a regional scale^67–71^. The coral skeletal δ^13^C record here is best interpreted as a proxy for changes in the coral’s autotrophy-heterotrophy balance and potentially a measure of coral response to bleaching. A combination of satellite products, climate and ocean reanalysis systems, buoys, and in-situ measurements are used to examine the role of atmosphere-ocean interactions in the modulation of reef-scale temperatures and coral thermal stress response at Ko Racha Yai. Our findings suggest that changes in tropical climate modes can modulate subsurface temperatures and the response of corals to heat stress through time at identified refuge sites, via large-scale atmosphere-ocean interactions, to an extent that was not previously known and that is unavailable from satellite observations and reef monitoring.

## Results and discussion

### Ko Racha Yai coral Sr/Ca as subsurface temperature record

**Fig. 2:**
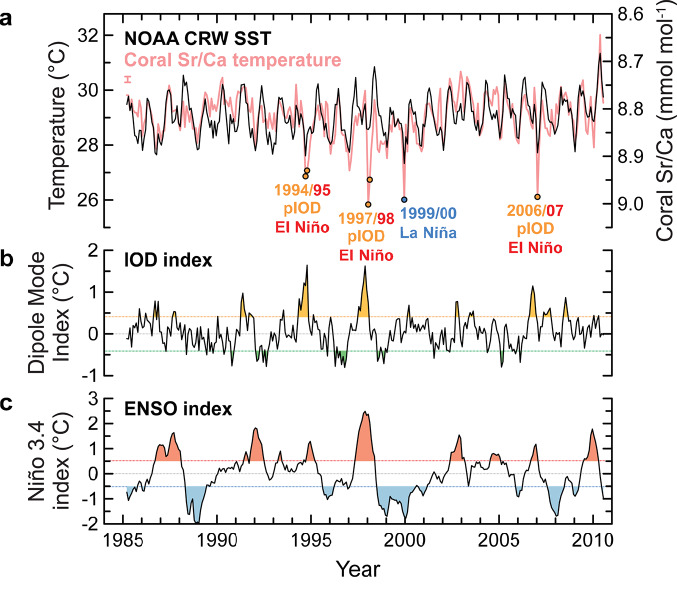
Coral Sr/Ca-based subsurface cooling events attributed to Indian Ocean Dipole (IOD) and El Niño-Southern Oscillation (ENSO) events. (**a**) Monthly Ko Racha Yai coral skeletal Sr/Ca and satellite sea surface temperature (NOAA Coral Reef Watch SST^25^) records scaled to their means, using a *Porites* coral Sr/Ca-temperature relationship of −0.0607 mmol mol^− 1^ per °C (ref.^52^). Coral Sr/Ca analytical error (1σ) is indicated. Extreme values in the monthly coral Sr/Ca record related to combined positive IOD (pIOD) and El Niño events and a La Niña event are highlighted (Supplementary Fig. 5). (**b**) Dipole Mode Index (DMI) as index for IOD^77^ with ±0.4°C threshold (coloured dashed lines)^78^. (**c**) Niño 3.4 index as index for ENSO^79^ with ±0.5°C threshold (coloured dashed lines)^80^. DMI and Niño 3.4 index calculated using ERSSTv5^72^.

The Ko Racha Yai coral provides a monthly-resolved proxy record of temperature variability in the southern Andaman Sea during 1985–2010 (Fig. 2a). X-radiography shows a clear skeletal pattern with annual high-density bands formed during the SW monsoon and low-density bands in the NE monsoon season, as inferred from the coral Sr/Ca-temperature annual cycles (Methods, Supplementary Fig. 1). Both X-radiograph and coral Sr/Ca annual cycles suggest that the analysed skeleton is well preserved with no evidence for early diagenesis. The mean annual cycle of coral Sr/Ca-temperature is 1.29 ± 0.40°C (±1σ) which broadly captures the satellite SST^25^ annual cycle of 1.72 ± 0.59°C (±1σ) (Supplementary Fig. 2a). The annual cycle of coral Sr/Ca has a similar double-peak pattern as observed in satellite SST and other gridded SST products^31,32,72,73^ (Fig. 2a, Supplementary Figs. 2a, 3), reflecting the seasonal forcing by the Asian monsoon^74^. Throughout the annual cycle, the coral Sr/Ca-temperature minimum occurs in January and the maximum in May, with a second less pronounced maximum occurring in November (Supplementary Fig. 2a). This pattern is consistent with satellite SST and Ko Racha Yai in-situ temperatures^12,43,44^, where minimum SST occurs in January and maximum SST in April-May (Supplementary Figs. 2a, 3). Coral Sr/Ca shows significant and strong inverse relationships with satellite SST and other SST products for monthly (*r* = −0.48 to −0.66, *p* < 0.01, *n* = 305) and annual average values (*r* = −0.52 to −0.68, *p* < 0.01, *n* = 26) (Table 1, Supplementary Tables 1, 2). Resulting coral Sr/Ca-SST relationships are mostly within the range reported in previous studies^52^ (−0.04 to −0.08 mmol mol^− 1^ per °C), and were tested using ordinary least squares (OLS), reduced major axis (RMA)^75^, and weighted linear squares (WLS)^76^ regressions (Table 1, Supplementary Tables 1, 2, Supplementary Fig. 4).

We note prominent discrepancies between the monthly records of Ko Racha Yai coral Sr/Ca-temperature and satellite SST during fall/winter in some years (Fig. 2a). Coral Sr/Ca suggests pronounced cooling events identified as extreme values (>8.93 mmol mol^− 1^ in monthly coral Sr/Ca) that are not evident in satellite SST, namely in 1994/95, 1997/98, 1999/00, and 2006/07 (Fig. 2a, Supplementary Fig. 5). Removing these cooling events lowers the absolute slope values of all coral Sr/Ca-SST relationships though they are mostly still within the range of previously reported relationships^52^ (Table 1, Supplementary Tables 1, 2). There are no notable changes in correlation coefficients and root mean square residuals (RSMR) across all calibrations (Table 1, Supplementary Tables 1, 2), indicating there are robust coral Sr/Ca-SST relationships with and without considering the cooling events. In some studies, such extreme deviations in Sr/Ca have been attributed to sampling issues^81^ or early diagenesis^82^, all of which we can exclude for our Sr/Ca record (Supplementary Note 1, Supplementary Figs. 6, 7). Additionally, we rule out proxy biases due to thermal stress bleaching, which can also be caused by cold temperature stress^83^, for the here identified cooling events. Our coral shows no signs of stress in the skeletal record through e.g., stress bands^84^ (Supplementary Note 1) and we conclude that the temperatures derived from Sr/Ca reflect in-situ temperatures and actual cooling events.

**Fig. 3:**
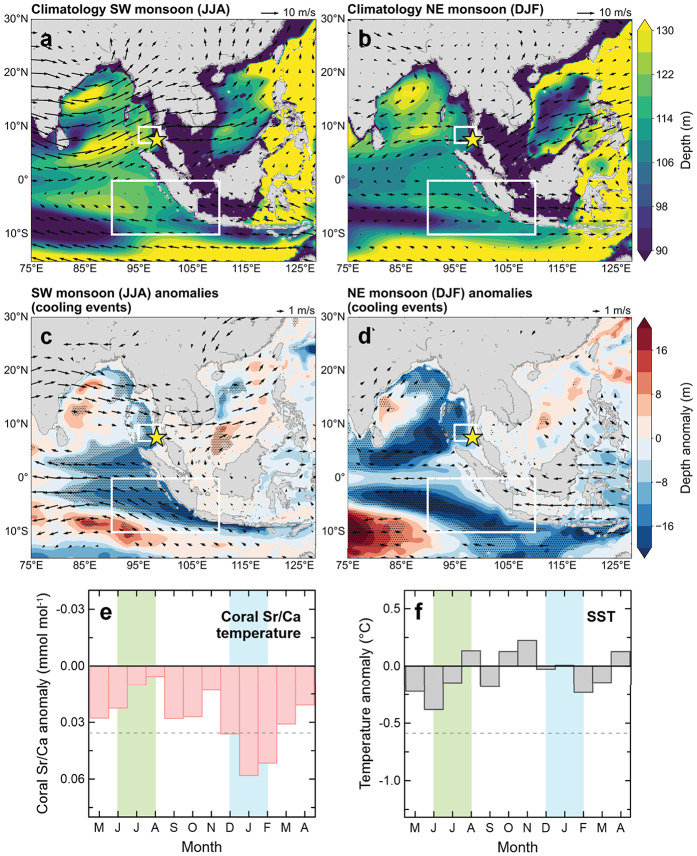
Seasonal characteristics of coral Sr/Ca-based subsurface cooling events. Climatology of wind vectors at 850 hPa level (ERA5^50^) and thermocline depth (depth of 20°C isotherm^45^, ORAS5^90^) for (**a**) southwest (SW) monsoon (June-July-August, JJA) and (**b**) northeast (NE) monsoon (December-January-February, DJF) seasons (1985–2010). Yellow star: Ko Racha Yai. Rectangles: Andaman Sea (7°N-10°N, 95°E-99°E) and equatorial eastern Indian Ocean (10°S-0°N, 90°E-110°E) domains. (**c**, **d**) Same as (**a**, **b**) but for composite anomalies of coral Sr/Ca-based subsurface cooling events (1994/95, 1997/98, 1999/00, 2006/07) (Fig. 2, Supplementary Fig. 5), significant at confidence levels 95% (thermocline depth, dots) and 80% (wind, black arrows) using a two-tailed Welch’s *t*-test. Monthly composite anomalies of Ko Racha Yai (**e**) coral Sr/Ca and (**f**) satellite sea surface temperature (SST) (NOAA CRW^25^) for cooling events. Vertical bars: approximate peaks of SW monsoon (green) and NE monsoon (blue) seasons. Horizontal dashed line: 1σ of mean satellite SST annual cycle scaled with coral Sr/Ca-temperature relationship of −0.0607 mmol mol^− 1^ per °C (ref.^52^). Anomalies are relative to climatology.

Comparisons with Indian Ocean Dipole (IOD) and El Niño-Southern Oscillation (ENSO) indices suggest the coral Sr/Ca-based cooling events at Ko Racha Yai coincide with years when a positive IOD event (pIOD) occurs in combination with an El Niño event in the tropical Pacific, such as in 1994/95, 1997/98, and 2006/07 (Fig. 2, Supplementary Fig. 5). A further cooling event is recorded in 1999/00, during a tropical Pacific La Niña event. The reconstructed cooling events occur during fall (September-October) in 1994/95, and winter (December-February) in 1997/98, 1999/00, and 2006/07 (Fig. 2a). We note that a potential minor cooling event in 1996/97 is rather an artefact of the linear trends (i.e., the year is not identified as an interannual climate event after detrending and removing low-frequency variability) and coincides with a negative IOD year (nIOD) (Supplementary Fig. 5). Our findings are unexpected as Ko Racha Yai in the southern Andaman Sea is located far north of the main area of IOD-induced SST anomalies in the equatorial eastern Indian Ocean (10°S-0°N, 90°E-110°E)^77,85^ (white rectangle, Fig. 3a–d). This is in line with the absence of significant correlations of Ko Racha Yai SST and the IOD index (Dipole Mode Index/DMI) in any season (Supplementary Fig. 8). Previous studies investigating the influence of IOD and ENSO on the Andaman Sea solely relied on satellite SST^86,87^. The cooling events captured by coral Sr/Ca are therefore interpreted to reflect substantially colder temperatures at depths where reefs are growing, which stay undetectable at the surface where satellite SST is recorded, mainly occurring in years characterised by combined pIOD and El Niño events. Together with observed enhanced upwelling^35^ and negative sea level anomalies^88^ in the Andaman Sea during pIOD events, the coral Sr/Ca-based cooling events are in line with what is typically observed further south, in the equatorial eastern Indian Ocean, the main IOD region^77,85^. We thus report a distinct IOD signal in a subsurface temperature proxy record in an area of the northeastern Indian Ocean which based on historical and satellite SST observations is normally considered too far north to be impacted by IOD events^89^.

**Table 1:** Ordinary least squares (OLS) regression equations and Pearson correlation coefficients, *r* (*r*^2^) between Ko Racha Yai coral Sr/Ca and satellite sea surface temperature (SST) (NOAA CRW^25^) for monthly and annual average (April-March) records (1985–2010) using all data, and with coral Sr/Ca-based cooling events excluded. Cooling events were identified as extreme values in the monthly coral Sr/Ca record (Fig. 2a, Supplementary Fig. 5).

Period	OLS regression equation	*r* (*r*^2^)	*p*	RMSR^b^	*n*
“Mean” equation^a^	Sr/Ca = −0.0607 × SST + 10.553				
All data (1985–2010)					
Monthly	Sr/Ca = −0.043 (± 0.003) × SST + 10.065 (± 0.082)	−0.66 (0.44)	<<0.01	0.034	305
Annual	Sr/Ca = −0.052 (± 0.011) × SST + 10.317 (± 0.33)	−0.68 (0.46)	<<0.01	0.023	26
Cooling events excluded (1985–2010)					
Monthly	Sr/Ca = −0.038 (± 0.003) × SST + 9.918 (± 0.075)	−0.65 (0.43)	<<0.01	0.03	298
Annual	Sr/Ca = −0.048 (± 0.011) × SST + 10.212 (± 0.307)	−0.68 (0.47)	<<0.01	0.021	26

^a^Mean of several published coral Sr/Ca-SST^52^.

^b^Root mean square residuals (RMSR) were calculated as the square root of the sum of all residual values.

The coral Sr/Ca-based cooling events at Ko Racha Yai are best explained by enhanced subsurface exposure to thermocline shoaling and LAIW, which transport colder subthermocline water to the reefs of the Andaman Sea^12,46^, but rarely to the sea surface where satellite SST is recorded. This can explain why the IOD signal is not evident in satellite products, such as NOAA Coral Reef Watch (CRW) SST^25^, but in coral Sr/Ca-reconstructed subsurface temperature (Fig. 2a,b). On the seasonal timescale the LAIW influence on Andaman Sea coral reefs is controlled by changes in thermocline depth modulated by upper ocean stratification^74^, which are influenced by local winds and the propagation of remote forcing (Kelvin waves and Rossby waves) from the equatorial Indian Ocean to the Andaman Sea^91,92^. Typically, the LAIW influence on the reefs is weaker during the SW monsoon season, and stronger during the NE monsoon^12,^^44,47^ (Fig. 3a,b). During the SW monsoon (June-August), surface waters are accumulated along the western side of the islands of the Andaman Sea by the southwesterly winds^44^. Furthermore, there are more downwelling Kelvin waves propagating from the equator to the Andaman Sea^91^. Both processes result in a deeper thermocline and thus a weaker LAIW influence on the reefs^12,47^ (Fig. 3a). Conversely, the NE monsoon (December-February) pushes surface waters away from the islands, and more upwelling Kelvin waves are associated with this season. This results in a shallower thermocline, well-stratified waters, and a stronger LAIW influence on the reefs^12^ (Fig. 3b). Importantly, during years with coral Sr/Ca-based cooling events, both monsoon seasons are characterised by easterly wind anomalies along the equator, in particular the critical SW monsoon season that is usually characterised by a deeper thermocline (Fig. 3c,d). This remote forcing results in the following changes in the Andaman Sea: more upwelling Kelvin waves^91^, shallower thermocline^45^, and thus enhanced LAIW^45^ cooling of subsurface reef waters, as recorded by coral Sr/Ca (Fig. 3e,f). In summary, our findings suggest that the monthly coral Sr/Ca record from the western side of Ko Racha Yai captures subsurface temperature variability at depths where coral reefs grow, modulated by thermocline shoaling coupled with LAIW that are extraordinarily prominent in the Andaman Sea. Satellite products commonly used in coral reef heat stress assessments such as NOAA CRW SST^25^ are unable to provide this information.

### Interannual to decadal changes of reef cooling in the Andaman Sea

**Fig. 4:**
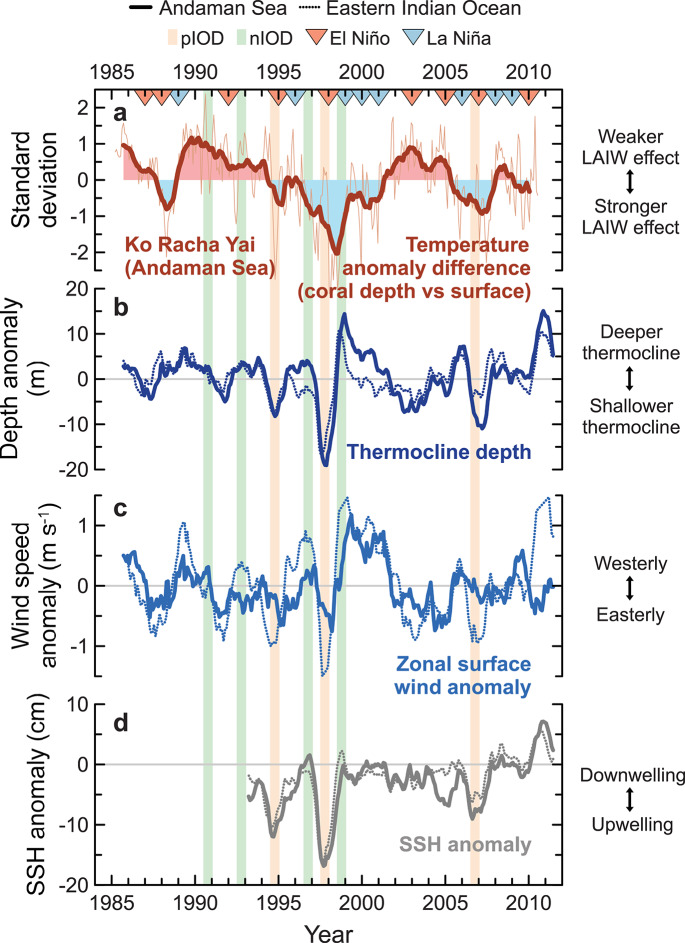
Andaman Sea and equatorial eastern Indian Ocean interannual to decadal variability. (**a**) Standardised temperature anomaly difference between surface and coral depth (~10 m) at Ko Racha Yai calculated by subtracting anomalies of NOAA CRW sea surface temperature^25^ (SST) from coral Sr/Ca temperature using a relationship of −0.0607 mmol mol^− 1^ per °C (ref.^52^) (mean subtracted and divided by standard deviation). Thin line: monthly record, thick line: 13-month moving average. (**b**) Thermocline depth anomaly (depth of 20°C isotherm^45^, ORAS5^90^) at buoy locations in the central Andaman Sea (9.6°N, 95.6°E)^93,94^ and equatorial eastern Indian Ocean (0°, 90°E)^95^ (Supplementary Fig. 10). (**c**) Zonal surface wind anomalies (ERA5^50^) over the Andaman Sea (7°N-10°N, 95°E-99°E) and equatorial eastern Indian Ocean (10°S-0°N, 90°E-110°E) (rectangles, Fig. 3a–d). (**d**) Sea surface height (SSH) anomaly from altimetry (7.75°N, 98.417°E) and tide gauge (7.83°N, 98.43°E) at Phuket (https://ccar.colorado.edu/altimetry). 13-month moving averages (b-d). Anomalies are relative to climatology (1985–2010). Vertical bars: positive Indian Ocean Dipole (pIOD, orange) and negative IOD (nIOD, green) events as indicated by both refs.^78,89^. Triangles: El Niño (red) and La Niña (blue) events^80^.

We explore potential changes of reef cooling on interannual to decadal timescales in the Andaman Sea driven by thermocline shoaling and LAIW. The 13-month moving averages of monthly satellite SST^25^ and coral Sr/Ca temperature at Ko Racha Yai indicate cooling periods at coral depth (~10 m) that are not observed in the satellite product, such as between ~1996 to ~2001 and ~2005 to ~2007 (Supplementary Fig. 9a,c). We further quantified these differences by subtracting the satellite SST anomalies from the coral Sr/Ca temperature anomalies, using standardised values (Fig. 4a, Supplementary Fig. 9b,d). Negative values are interpreted as colder coral temperatures compared to the surface due to thermocline shoaling and stronger LAIW influence at coral depth. Conversely, positive values represent a deeper thermocline and a weaker LAIW influence on the reef. We note that this interpretation and result is independent of the coral Sr/Ca-temperature relationship used (Supplementary Fig. 9).

The reconstructed interannual to decadal changes in reef cooling at Ko Racha Yai, driven by thermocline shoaling and LAIW, are consistent with large-scale thermocline variations in the Andaman Sea and equatorial eastern Indian Ocean (Fig. 4a,b). Thermocline depth in both regions is largely influenced by IOD and ENSO via zonal wind changes, which induce an anomalous remote forcing from the equatorial region to the Andaman Sea^91,96,97^. This can be seen in the agreement between anomalies of thermocline depth, zonal winds, and sea surface height (SSH) in reanalysis data^90^ and buoy measurements^93–95^ for both regions (Fig. 4, Supplementary Fig. 10). It appears that a combined pIOD and El Niño event (e.g., 1997/98) is related to a longer time interval of stronger upwelling influence on the reef (e.g., ~1996 to ~2001) (Fig. 4a). During combined pIOD and El Niño events, a weakening of the Walker circulation induces subsidence and anomalous easterly winds along the equator, resulting in more upwelling Kelvin waves, enhanced upwelling, and a shallower thermocline in both regions^35,85,91^ (Fig. 4). The peak of the period of strong reef cooling broadly coincides with the typical peak of LAIW in the Andaman Sea around January-March^12^ (e.g., 1998), lagging the peak of thermocline shoaling that occurs during early winter by ~1–2 months (Fig. 4a,b).

**Fig. 5:**
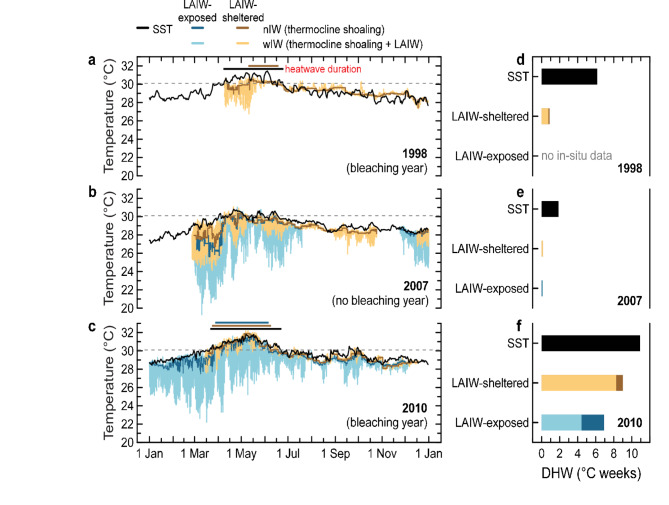
Drivers of reef cooling in the southern Andaman Sea. Ko Racha Yai daily satellite sea surface temperature (SST) (NOAA CRW^25^) and sub-daily in-situ temperature measurements at western (large-amplitude internal waves (LAIW)-exposed) and eastern (LAIW-sheltered) sides of southern Andaman Sea islands for years with (1998, 2010) and without bleaching (2007) (see Fig. 1 for locations). (**a**) 1998 (Surin Island), 15 m depth^46^. (**b**) 2007 (Miang Island), 20 m depth^47,48^. (**c**) 2010 (Tachai Island, LAIW-exposed side; Surin Island, LAIW-sheltered side), 15 m depth^12^; extreme LAIW-exposed/-sheltered sites to reveal full range of heat stress dampening. In-situ temperature “with internal waves” (wIW) reflects effects of thermocline shoaling and LAIW, “no internal waves” (nIW: low-pass filtered wIW) reflects thermocline shoaling only^10^. Horizontal bars: heatwave duration in 1998 and 2010 at surface (reef depth) inferred from SST (nIW) >30.1°C bleaching threshold^100^ (horizontal dashed line). (**d**–**f**) Corresponding cumulative Degree Heating Week (DHW)^10^ values at surface (reef depth) based on SST (nIW, wIW), revealing relative contributions of different drivers in abating heat stress at reef depth relative to surface. Substantial reef cooling in 1998 was mainly by thermocline shoaling (DHW value reduced from ~6 to ~1°C weeks), with only minor additional contribution by LAIW (**d**). Less intense reef cooling in 2010 (DHW value reduced from ~11 to ~9°C weeks) had a larger contribution by LAIW, especially at LAIW-exposed site (**f**). Substantial (1998) and less intense (2010) reef cooling for heat stress reduction are consistent with our reconstruction (Fig. 4a).

At the regional scale, the reconstructed interannual to decadal changes in reef cooling at Ko Racha Yai (Fig. 4a) are supported by rare in-situ temperature measurements. Along the Thai islands of the southern Andaman Sea, a strong influence of upwelling on shallow reef habitats (7–20 m) through thermocline shoaling and stronger LAIW activity can be inferred for 1998 (ref.^46^) and 2007 (ref.^47^) (Fig. 5, Supplementary Fig. 11) at depths where our coral grew (~10 m), consistent with our reconstruction (Fig. 4a). Following the approach by Wyatt et al.^10^, we consider in-situ temperature at reef depth as reflecting the effects of thermocline shoaling and LAIW (“with internal waves”, wIW), whereas the low-pass filtered version of wIW reflects the effect of thermocline shoaling only (“no internal waves”, nIW) (Fig. 5). The marked difference between SST and reef temperature without LAIW (nIW) in 1998 and 2007 suggests prominent cold-water intrusions by thermocline shoaling during both years, in addition to the influence of LAIW (Fig. 5a,b). Further support for enhanced upwelling during 1998 and 2007 comes from colder temperatures across 10 to 145 m depth relative to 2010 inferred from logger, mooring, and CTD data throughout the region (Supplementary Fig. 11). This observational evidence is also in line with the two most prominent coral Sr/Ca-based cooling events occurring during combined pIOD and El Niño conditions (1997/98, 2006/07) (Fig. 2). The results suggest the Ko Racha Yai subsurface temperature proxy record reflects large-scale changes in reef cooling across the southern Andaman Sea on interannual to decadal timescales, driven by thermocline shoaling and LAIW. Large-scale atmosphere-ocean processes, together with an anomalously shallower thermocline during both monsoon seasons that characterise years with coral Sr/Ca-based cooling events (Fig. 3), may have supported the prolonged subsurface cooling of shallow reef habitats between ~1996 to ~2001 and ~2005 to ~2007 (Fig. 4), undetectable at the surface where satellite SST is recorded. We note that these sustained periods of reef cooling can encompass ~monthly episodes of weaker reef cooling, which usually occur during the SW monsoon season.

During El Niño years not accompanied by pIOD events, similar but weaker processes occur^96^, resulting in less thermocline shoaling and a relatively weaker LAIW influence on the Andaman Sea reefs (Fig. 4). This is supported by no notable differences between SST and nIW temperature observed during the 2009/10 El Niño along the southern Andaman Sea islands (Fig. 5c). Opposite processes occur during nIOD and La Niña events, with a strengthening of the Walker circulation leading to anomalous westerly equatorial winds, more downwelling, deeper thermocline^98^, and a weaker LAIW influence (Fig. 4). A weaker upwelling influence on the reefs during La Niña is supported by in-situ measurements at Ko Racha Yai that indicate similar temperatures at surface and subsurface during the 2010/11 La Niña, compared to the preceding 2009/10 El Niño when subsurface temperature was up to ~1°C colder at 15 m depth (Supplementary Fig. 3). The latter implies even colder subsurface temperatures may have occurred during the peak of the reconstructed ~1996–2001 and ~2005–2007 periods of prominent reef cooling at Ko Racha Yai as the 2009/10 El Niño actually occurred during an inferred interval of relatively weak reef cooling (Fig. 4a), in line with in-situ observations along the southern Andaman Sea islands (Fig. 5). However, an exception may have occurred during the 1999/00 La Niña, one of the four prominent coral Sr/Ca-based cooling events (Fig. 2a). The 1999/00 La Niña event is part of a triple-dip La Niña (1998–2001) (Fig. 2c) characterised by prominent reduced surface heating due to a westward shift of the Walker circulation^99^. Together with a shoaling of the thermocline after the 1998/99 La Niña (Fig. 4b) this may have enhanced reef cooling in 1999/00. Overall, our results suggest that reef cooling by thermocline shoaling and LAIW activity at Ko Racha Yai, a thermal refuge site in the southern Andaman Sea, is not constant through time but changes in strength on interannual to decadal timescales. These changes are mainly influenced by thermocline and wind variations modulated by IOD and ENSO via remote forcing from the equatorial eastern Indian Ocean.

### Coral thermal stress response signal in Ko Racha Yai skeletal δ^13^C record

**Fig. 6:**
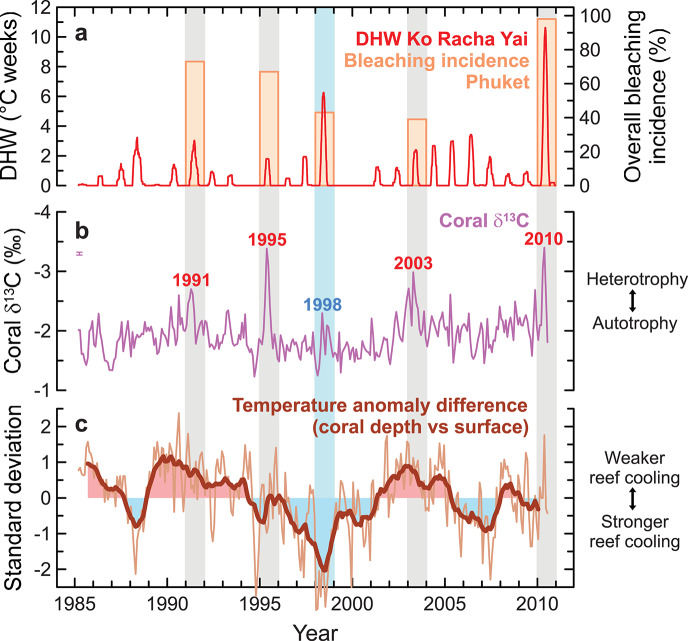
Ko Racha Yai coral stress response during major southern Andaman Sea bleaching events. (**a**) Degree Heating Week (DHW) at Ko Racha Yai (red line) and incidence of percentage bleaching in massive coral colonies at Phuket (~23 km north of Ko Racha Yai)^100^ (orange bars). DHW calculated from daily NOAA CRW sea surface temperature (SST)^25^, identified when daily SST is above a bleaching threshold of 30.1°C over a 12-week moving window^100^. Overall bleaching incidence shown is the sum of totally and partially bleached corals^100^. (**b**) Monthly Ko Racha Yai coral skeletal δ^13^C record. Error bar: analytical error (1σ). (**c**) Standardised temperature anomaly difference between surface and coral depth (~10 m) at Ko Racha Yai (see Fig. 4a for details). Monthly values (thin line) and 13-month moving average (thick line) are shown. Years with observed major bleaching events in the southern Andaman Sea of Thailand are indicated (vertical bars)^46,100^. The 1998 event is highlighted (blue).

Major heatwaves were identified for the Andaman Sea region based on SST for the years 2010, 2003, 1998, 1995, and 1991 that were accompanied by varying degree of bleaching incidence in shallow reef flat habitats^100^, confirming the heating of surface waters (Fig. 6a). We assessed the coral skeletal δ^13^C signal as it provides us insights into the coral metabolic balance during such extreme events. The coral δ^13^C annual cycle at Ko Racha Yai resolved at monthly resolution reflects the transient contributions of various parameters throughout the year (Supplementary Fig. 2, Supplementary Note 2). In addition, the coral δ^13^C record reveals pronounced excursions towards more negative values during the years 2010, 2003, 1995, and slightly less pronounced in 1991 (Fig. 6b). Interestingly, these excursions coincide with the reported moderate to severe bleaching events in the southern Andaman Sea observed in Phuket^46,100,101^, and are accompanied by elevated Degree Heating Week (DHW) values at Ko Racha Yai derived from SST^25^ (Fig. 6a,b). The pronounced negative excursions of skeletal δ^13^C are probably best explained by the coral’s reduced photosynthetic activity^67–71^ during heat stress events, due to decreased activity or partial loss of its endosymbiotic algae^102,103^. However, a higher contribution of heterotrophy can shift coral δ^13^C in the same direction and both processes, decreased autotrophy (i.e., less photosynthesis) and increased heterotrophy, would shift skeletal δ^13^C to more negative values^67–71,104^ (Supplementary Note 2). We here speculate that the coral actively compensates the loss of autotrophic energy supply through its symbionts by increased heterotrophy and feeding on zooplankton during heat stress^18–20,105,106^. Such increased heterotrophy is in line with enhanced particle flux during LAIW activity and the observed higher metabolic and trophic plasticity in corals from LAIW-exposed western sides of the islands in the southern Andaman Sea^17,107,108^. Furthermore, this contributes to increased energy reserves^17,108^ and potentially can help corals to endure and recover quickly from symbiont loss^109^.

This is in line with the observation that the coral skeleton does not reveal any anomalous growth patterns or stress bands associated with the negative skeletal δ^13^C excursions during the reported heat stress and bleaching events in the Phuket region^46,100,101^. X-ray imaging indicates continuous, undisturbed growth, and a well-developed annual skeletal density-banding pattern throughout the entire record (Supplementary Fig. 1). Further, the annual growth rate shows no anomalous decrease during or after years with observed heat stress events (Supplementary Fig. 12), supporting our finding that thermal refuges exist due to cooling in deeper parts of the reef (Figs. 5, 6c, Supplementary Fig. 11). Thus, we speculate that the colony did not show a strong bleaching response, which is consistent with the observation that substantially reduced growth rates and stress bands in southern Andaman Sea corals are often visible only in fully bleached but not partially bleached (i.e., pale) colonies^110^.

Our skeletal δ^13^C-based results from Ko Racha Yai provide insights into a coral’s metabolic activity during repeated heat stress events without being masked by so-called kinetic isotope effects^111^, due to the absence of reduced growth and/or calcification rates that are commonly observed during such events^112^. We suggest that the excursions towards more negative coral δ^13^C values in 2010, 2003, 1995, and 1991 may represent subtle responses to heat stress events that are to some extent abated by subsurface cooling through thermocline shoaling and LAIW at the depth of the coral, potentially reflecting changes in the coral’s autotrophy-heterotrophy balance towards more heterotrophy. Our results are consistent with multiple evidence that heterotrophic feeding and/or shifts to more heterotrophy can improve coral resilience during bleaching^18–20,113^. Thus, our inferred shifts in the autotrophy-heterotrophy balance during multiple heat stress events suggest a physiological response that may act as further mechanism to support coral thermal resistance at this Andaman Sea refuge site, alongside with environmental memory by LAIW-induced cooling^37^ and a heat-tolerant microbiome^114, 115^. However, given the various controls on coral skeletal δ^13^C (refs.^67–71^), some caution is required when interpreting it as a universal bleaching proxy.

Interestingly, in 1998 the coral skeletal δ^13^C record does not show an excursion towards more negative values, which may suggest no metabolic imbalance and only minor or no bleaching during this reported heat stress event (Fig. 6a,b). This is strongly in line with our reconstruction of interannual to decadal changes in upwelling influence on Andaman Sea reefs based on coral Sr/Ca, which clearly underscores that the 1998 bleaching event occurred during a period of intense subsurface reef cooling by thermocline shoaling and LAIW (~1996 to ~2001) (Fig. 6c). Consequently, we assume the colony’s photosynthetic activity was not affected during this event, because at the depth it is growing (~10 m) thermal stress was mitigated by enhanced advection of colder subthermocline water to the subsurface. Thus, the colony was protected from the heat stress at the sea surface indicated by the NOAA CRW SST product^25^, reaching DHW values above 6°C weeks at Ko Racha Yai (Fig. 6). Our results thus provide another line of evidence and explanation for the rather mild or minor bleaching in the southern Andaman Sea during 1998 (refs.^46,101^) despite being on a global scale a massive bleaching event^4,5^. The findings underscore that deeper reefs were protected at this thermal refuge site, while reef flat coral populations did bleach^100^ as they experienced a strong heating. The combined results demonstrate that coral Sr/Ca-based subsurface temperature reconstructions and simultaneous skeletal δ^13^C analysis can fill the knowledge gap regarding underwater thermal environmental conditions and coral’s metabolic response when in-situ temperature and reef monitoring are lacking. However, given the various controls on coral skeletal δ^13^C (refs.^67–71^), the absence of negative excursions (e.g., in 1998) does not uniquely constrain coral metabolic state or bleaching severity.

We note that an enhanced upwelling influence as inferred for 1998 should be generally accompanied by increased inorganic nutrients^108^ thus resulting in more negative seawater δ^13^C_DIC_ and more heterotrophy (i.e., more negative skeletal δ^13^C)^68,116^, which is not observed. However, autotrophy could still be the most dominant driver for coral δ^13^C as the nutrients could benefit both coral host and symbiont^117^. This is supported by higher light availability due to pronounced negative SSH anomalies and reduced cloud cover associated with the combined pIOD and El Niño 1997/98, potentially outpacing the opposite effect of higher turbidity during this reconstructed strong upwelling period (Supplementary Note 3, Supplementary Fig. 13). These factors together with the mild or minor bleaching in 1998 may mask any enhanced feeding by the coral, resulting in the maintenance of more positive δ^13^C values. This is different to the preceding bleaching events in 1991 and 1995 that were described as more severe^46,100,101^. While these two events were accompanied by substantially lower SST-based DHW values relative to 1998, they occurred during a period of relatively weak influence of reef cooling by thermocline shoaling and LAIW, which potentially contributed to the rather intense bleaching in these years (Fig. 6).

The different response in coral skeletal δ^13^C in 1998 compared to the other bleaching events highlights that reef cooling by upwelling influence controls the intensity of thermal stress mitigation in the region through time (Fig. 6). However, we find that the relative contribution of thermocline shoaling and LAIW to thermal stress mitigation can also determine its spatial distribution, as inferred from in-situ temperature measurements. Reef cooling can be limited to the LAIW-exposed western side of the southern Andaman Sea islands such as in 2010, or covering a larger spatial extent by also protecting the LAIW-sheltered eastern side of islands like in 1998 (Figs. 5, 6). In 2010, it was solely the LAIW cooling that occurred and mainly protected corals in LAIW-exposed western sides^12,21^, as supported by similar nIW temperatures between LAIW-exposed and LAIW-sheltered sides (Fig. 5c,f). However, in 1998, when the thermocline shoaled substantially as inferred from coral Sr/Ca and in-situ measurements, the latter also indicate LAIW activity on sheltered sides was stronger compared to 2010 (Figs. 5, 6). Thus, it can be expected that in LAIW-exposed sides in 1998, the LAIW cooling must have been even stronger compared to 2010, and further amplified by thermocline shoaling. This is in line with the absence of metabolic imbalance suggested by skeletal δ^13^C of our coral at the LAIW-exposed side in 1998, and the observed mild bleaching in the region^46,101^. Thermocline shoaling may be even more important for heat stress reduction during bleaching events compared to LAIW. At the LAIW-sheltered side, in-situ measurements indicate thermocline shoaling contributes to heat stress reduction by 17.5% (2010) and 85.7% (1998) while the contribution of LAIW is 6.8% (2010) and 2.2% (1998) only (Fig. 5d,f). Both processes may have protected the Andaman Sea reefs to certain extent during past heatwaves, but the focus of recent research was mostly on the role of LAIW in reef cooling. Therefore, cooling through both LAIW and thermocline shoaling may contribute to the diversification of thermal refugia in the Andaman Sea region and the latter may be an overlooked process even reaching into shallow reef habitats.

In summary, the coral skeletal δ^13^C record adds another important facet to the coral Sr/Ca-based thermal reconstruction of subsurface conditions at Ko Racha Yai by providing insights into the potential coral response during heat stress in the southern Andaman Sea. The coral δ^13^C signal confirms our identification of a prominent period of reef cooling at the subsurface that accompanied a past heatwave event at the surface in 1998, providing protection from heat stress and potentially preventing strong bleaching. While during heat stress events that were not fully buffered by subsurface reef cooling (1991, 1995, 2003, 2010), the coral likely experienced to some extent symbiont loss that was compensated by heterotrophic feeding, leading to clear negative excursions in skeletal δ^13^C. We do note, however, that coral δ^13^C can be influenced by various parameters^67–71^ at Ko Racha Yai (Supplementary Note 2, 3). Hence, coral δ^13^C cannot be interpreted exclusively as a bleaching proxy, yet our results suggest that metabolic changes are likely the dominant control on Ko Racha Yai coral δ^13^C during heat stress events in the southern Andaman Sea. Further, the relative contributions of metabolic processes for the coral δ^13^C excursions, especially loss of symbiont versus increased feeding, warrant further investigations. Finally, we note that our study is based on a single colony, and that replication across multiple colonies within a single reef, or different reefs of different islands, in future studies would help to assess how representative our findings are of broader scale physiological responses of southern Andaman Sea corals to heat stress.

## Conclusions

Our results based on combining coral skeletal geochemical and isotopic records from Ko Racha Yai with climate, ocean, and environmental observations provide novel insights into the underlying drivers for subsurface reef cooling in the Andaman Sea, a prominent thermal refuge site in the northeastern Indian Ocean^12,51,118^. We find that on interannual to decadal timescales, tropical climate modes, such as IOD and ENSO, control the intensity and spatial distribution of thermal stress mitigation by thermocline shoaling, coupled with intensification of LAIW (summarised in Supplementary Fig. 14). This suggests that this refuge site from global warming does not always provide reliable protection of coral reefs from marine heatwaves as previously assumed. Satellite products commonly used in heat stress assessments for coral reefs such as NOAA CRW SST^25^ are unable to provide this information, as they do not record reef-scale temperatures at depth where most corals grow. Further, coral reef monitoring is usually unable to detect historical, population-scale differences in the thermal stress response, such as the subtle response to minor heat stress reflecting changes in the coral’s autotrophy-heterotrophy balance towards more heterotrophy, during periods when reef cooling by thermocline shoaling and LAIW was relatively weak.

## Methods

### Coral core collection

The coral core (TH3A) was drilled on 19 October 2011 at Siam Bay on the north coast of Ko Racha Yai (7°36’50”N, 98°22’8.54”E) (Thailand) in the southern Andaman Sea (Fig. 1a). The core was taken from a ~2.8-m-high massive *Porites lobata* colony growing at ~10 m water depth. The 5.5-cm-diameter core was drilled vertically from the top of the colony along the main axis of coral growth using a diver-operated pneumatic drill. The coral species was identified microscopically by using Corals of the World^119^ as the primary taxonomic reference.

Ko Racha Yai is an offshore island with reefs extending to a depth of ~15–20 m (ref.^46^). The reefs in Siam Bay do not form a significant reef-flat feature. The shore declines gently to the fore reef in ~10 m depth^120^. Thus, Siam Bay faces the Andaman Sea without any barrier and likely reflects open-ocean conditions. The bay is more or less open to the west of Ko Racha Yai, making it influenced by the eastward propagating LAIW^12,43^. The bay is also exposed to high hydraulic energy as both the LAIW and surface waves come from the same westerly direction^44,120^.

### Coral microsampling

The core was cut into two halves and 7-mm thick slabs were sliced from each half. The slabs were cleaned with a water nozzle/jet immediately after cutting for 3 minutes using tap water to avoid any powder from the cutting process settling within the pore spaces. Slabs were soaked in an ultrasonic cleaner for 12 minutes and then dried in an oven at 50°C overnight. Each slab was X-rayed at 50 kV for 5 minutes using an X-ray cabinet Faxitron 43855A (Hewlett-Packard) at MARUM – Center for Marine Environmental Sciences, University of Bremen, Germany to reveal the skeletal density banding pattern of alternating high- and low-density band pairs, which each pair representing one year of coral growth (Supplementary Fig. 1). The X-radiographs were used to determine (1) the optimum set of slabs and (2) the optimum path for microsampling on the selected set of slabs, avoiding areas affected by bioerosion and potential early diagenesis, as well as an additional guide for constructing the age model.

Microsampling was conducted using a WABECO F1210 milling machine that has a precision of 0.01 mm and is adjustable in XYZ directions applying continuous spot-sampling following Felis et al.^61,63,68,121^. Coral slabs were microsampled using dental drill bits with 0.8- and 0.9-mm diameter, depending on the coral growth rate as inferred from the annual density banding pattern, resulting in an average resolution of 17 samples/year. The microsampling depth was ~4 mm. Microsampling followed the main axis of coral growth, prioritising the centres of single fans of corallites and corallite growth directions parallel to the slab surface, to ensure reliability and chronological accuracy of the geochemical records^68,81,121^. Control X-radiographs of microsampled slabs show that the microsampling transects follow precisely the main growth axis of single fans of corallites (Supplementary Fig. 1).

### Geochemical analyses

Coral Sr/Ca and Mg/Ca were measured at MARUM, University of Bremen, Germany. The 0.35–0.65 mg of coral skeletal powder were weighed on a Sartorius SE2 microbalance using aluminium microcups and then instantly transferred to 7 mL vials. Aluminium microcups were reweighed after transfer and if there was no (≤0.005 mg) residual powder on them then the microcups were reused. Coral powder was dissolved in the vial with 3.5–6.5 mL of 2% HNO_3_ (30 mL 65% subboiled HNO_3_/1 L Milli-Q water) to get a nominal concentration of 40 ppm Ca (real concentration is ~38 ppm Ca, ~5% of coral powder was lost during transfer to vials). Coral Sr/Ca and Mg/Ca were measured using simultaneous axial Inductively Coupled Plasma Optical Emission Spectrometry (ICP-OES) (Agilent 720 series) with a twister spray chamber and a conical nebuliser. The Sr/Ca and Mg/Ca ratios of each of three replicate simultaneous Sr, Mg, and Ca measurements were averaged. The element/Ca ratio standard deviation of three replicates is typically better than 0.35%. Instrumental drift was corrected by measuring an inhouse coral standard before and after every sample assuming stable Sr/Ca and Mg/Ca ratios in the inhouse standard. The inhouse standard is a dissolved real coral powder with a nominal Sr/Ca value of 9.29 mmol mol^− 1^ and Mg/Ca value of 4.33 mmol mol^− 1^. Interlaboratory comparability was assured by measuring the JCp-1 coral reference material^122^ for at least every 50 samples. The JCp-1 reference composition reported in this study is 8.921 ± 0.007 mmol mol^− 1^ for Sr/Ca and 4.151 ± 0.009 mmol mol^− 1^ for Mg/Ca. These represent the average value of ten Sr/Ca and Mg/Ca analyses of splits of JCp-1 powder that were treated like samples during the course of this study.

Coral δ^13^C was measured at MARUM, University of Bremen, Germany on a Finnigan MAT 252 gas isotope ratio mass spectrometer connected to a Kiel III automated carbonate preparation device. Isotopic values are reported relative to the Vienna Peedee Belemnite (VPDB) reference standard. The instrument was calibrated against the inhouse standard (ground Solnhofen limestone), which then was calibrated against the NBS 19 calcite. Reproducibility from replicate measurements of the inhouse carbonate standard over the measurement period is better than ±0.05‰ (1$\sigma$) for δ^13^C.

### Coral chronology

For chronology construction, the annual cycles in the monthly NOAA CRW SST, also known as CoralTemp version 3.1 (ref.^25^), centred at 7.625°N and 98.375°E were used as target (Supplementary Fig. 2a) for setting tie points for the coral Sr/Ca record. CRW SST has the highest spatial resolution (0.05° x 0.05°) in comparison with other gridded SST products^31,32,72,73^, and is used for coral bleaching alerts and heat stress monitoring by NOAA^25^. NOAA CRW SST also has the strongest correlation with in-situ data of near-surface temperature at Ko Racha Yai^43^ compared to other gridded SST products (Supplementary Fig. 3, Supplementary Table 3).

The coral chronology was constructed by setting the coral Sr/Ca maximum, minimum, second maximum, and second minimum of any given year to the corresponding SST minima and maxima in the NOAA CRW product (Supplementary Fig. 2a) using the QAnalySeries software^123^. Additional inflection points were tied when the variations of coral Sr/Ca were represented by the SST. Subsequently, a linear interpolation was done between these tie points to 12 equally spaced values per year, resulting in a monthly resolution for coral Sr/Ca, δ^13^C, and Mg/Ca records. Mean annual values were calculated by averaging monthly data from April to March in the following year (“tropical year”) to avoid dividing interannual events, like El Niño, into different years^124^.

The annual cycle in coral Sr/Ca was used to count annual layers in the coral, supported by the clear annual skeletal density-banding pattern, following established methods^57,59–61,63–65,68,121,125,126^ (Supplementary Fig. 15). High-density bands are precipitated during the SW monsoon and low-density bands in the NE monsoon season, which is in line with previous work on *Porites* corals at Ko Racha Yai^127^. The core top was set to September 2011, as the core was drilled in mid-October 2011 and consequently, the skeleton of that month had not been fully precipitated. Further, due to the presence of a layer with anomalously high coral Mg/Ca (~9–18 mmol mol^− 1^) at the top of the core located below the coral’s tissue layer (Supplementary Figs. 1, 16), and a disturbed coral Sr/Ca annual cycle throughout both layers, the time interval from September 2011 to August 2010 was not used for the coral record. Tissue layers contain organic material that may disturb the SST-driven Sr/Ca annual cycle recorded by the coral, as reported in previous studies^128,129^. The layer with anomalously high Mg/Ca ratios below the tissue layer corresponds to an area affected by bioeroders, as evident in high-density microboring traces in the X-ray image (Supplementary Fig. 1). Bioerosion by microborers, such as endolithic algae, has been reported to affect the geochemistry of coral skeletons^130^, and may result in a disturbed coral Sr/Ca-SST annual cycle as well. However, we note that the layer of microbioerosion is not identical with the greenish layer typically observed near the top of modern coral cores, which is usually attributed to endolithic algae activity. In our Ko Racha Yai coral core, this greenish layer is located even below the layer characterised by microbioerosion traces. Accordingly, the Ko Racha Yai coral Sr/Ca, δ^13^C, and Mg/Ca records presented in this study cover the period from July 2010 back to March 1985, which is the starting year of the NOAA CRW SST product that also best reflects local temperature logger data.

### Coral growth rate

The annual coral growth rate was calculated from the distance between the coral Sr/Ca maximum in a given year to the coral Sr/Ca maximum in the following year, i.e., from winter (~January) to the following winter. The growth rates range from 0.7 to 1.9 cm year^− 1^ with a mean of 1.1 cm year^− 1^ (1986–2010) (Supplementary Fig. 12). Thus, the growth rates are higher than the critical threshold of 0.6 cm year^− 1^, where kinetic isotope effects were reported to influence coral isotopic values^111,125^. The mean coral growth rate of 1.1 cm year^− 1^ is broadly comparable with previous multi-colony studies on *Porites* spp. at Ko Racha Yai, reporting mean growth rates of 1.7 cm year^− 1^ (*n* = 14, 1984–1986)^120^ and 1.5 cm year^− 1^ (*n* = 9, 2003–2005)^131^. There are weak but non-significant correlations between the annual growth rate and the mean annual coral Sr/Ca (*r* = −0.14, *p* > 0.05, *n* = 25) and coral δ^13^C (*r* = −0.2, *p* > 0.05, *n* = 25) values. This suggests there are no growth effects on the coral Sr/Ca and δ^13^C records. Thus, these proxies are primarily driven by climatic and environmental changes on interannual timescales.

## Supplementary Information

Below is the link to the electronic supplementary material.

Supplementary Material 1

## Data Availability

Coral data that support the findings of this study have been deposited in PANGAEA with the identifier https://doi.org/10.1594/PANGAEA.978488^132^. Other data relevant to this study can be downloaded from the websites listed: NOAA CRW SST^25^ (https://oceanwatch.pifsc.noaa.gov/erddap/griddap/CRW_sst_v3_1_monthly.html); ERA5 wind^50^ (10.24381/cds.6860a573); ORAS5 depth of 20°C isotherm^90^ (10.24381/cds.67e8eeb7); SSH anomaly (https://ccar.colorado.edu/altimetry/); buoy temperature data^93–95^ (10.17605/OSF.IO/NZQ2C, https://www.pmel.noaa.gov/tao/drupal/disdel/); OISSTv2^31^, OISSTv2.1^32^, HadISST^73^, ERSSTv5^72^, and FRESCO v6 surface solar irradiance^133^ (https://climexp.knmi.nl/); SeaWIFS chlorophyll-*a*^134^ (https://oceanwatch.pifsc.noaa.gov/erddap/griddap/sw_chla_monthly_2018_0.html); NOAA NCAR OLR^135^ (https://psl.noaa.gov/data/gridded/data.olrcdr.interp.html).
