# Supplementary material for: Tropical climate modes control strength and distribution of thermal stress mitigation in a coral reef refugia
Source: Sci Rep. 2026 Jun 21;16:19757. doi: 10.1038/s41598-026-52941-6 (PMC13316109; doi:10.1038/s41598-026-52941-6)
Supplement: Supplementary file 1 — Supplementary Material 1 [file 41598_2026_52941_MOESM1_ESM.pdf]

Supplementary Information for:

Tropical climate modes control strength and distribution of thermal stress mitigation in a coral reef refugia

Hana Camelia[1]*, Thomas Felis[1]*, Jessica A. Hargreaves[1], Martin Kölling[1], Sander Scheffers[2], Suchana Chavanich[3], Chalermrat Sangmanee[4] & Marlene Wall[5]

[1]MARUM – Center for Marine Environmental Sciences, University of Bremen, 28359 Bremen, Germany

[2]Oceans Institute, The University of Western Australia, Perth 6009, Australia

[3]Department of Marine Science, Faculty of Science, Chulalongkorn University, Bangkok 10330, Thailand

[4]Department of Marine and Coastal Resources, Phuket Marine Biological Center, Phuket 83000, Thailand

[5]GEOMAR Helmholtz Centre for Ocean Research Kiel, 24148 Kiel, Germany

*Corresponding author at: MARUM – Center for Marine Environmental Sciences, University of Bremen, 28359 Bremen, Germany

E-mail address: hcamelia@marum.de (H. Camelia), tfelis@marum.de (T. Felis)

Supplementary Note 1

Ko Racha Yai coral Sr/Ca-based cooling events

We rule out the possibility of sampling issues and early diagenesis in causing the pronounced cooling events in the Ko Racha Yai coral Sr/Ca record (Fig. 2a). A bias towards apparently colder temperatures in coral geochemical proxies can result from microsampling along the 'valley' of *Porites* corallites[1,2] or in areas of disorganised skeletal structure[3]. However, our X-radiography clearly shows that we microsampled along the main growth axis following the centre of individual corallite fans (the 'bump') that have clear skeletal structure (Supplementary Fig. 1), i.e., corallite orientation is parallel to the slab surface. Secondary aragonite has also been associated with a bias towards apparently colder temperatures in coral Sr/Ca[4,5] but we do not detect any anomalous high-density patches on our X-radiography that might indicate the presence of substantial secondary aragonite filling of skeletal pores[5,6]. Our Ko Racha Yai coral also reveals clear, undisturbed annual cycles in coral Sr/Ca (Fig. 2a, Supplementary Fig. 2a), and none of our coral Sr/Ca and Mg/Ca values encounter the broadly defined diagenetic aragonite field (Supplementary Fig. 6). Further, the scanning electron microscope (SEM) analysis along the coral slab used for geochemical analysis does not reveal any secondary aragonite or calcite cements in the skeletal pore spaces, resulting in excellent preservation of primary porosity (Supplementary Fig. 7). Thus, our Ko Racha Yai modern coral can be considered as well-preserved, as well as suitable for monthly-resolution coral geochemistry of the skeleton, and is not affected by early diagenetic alteration.

Another possible source for an apparent cold bias in the coral Sr/Ca-temperature proxy could be cold stress, which can also cause coral bleaching[7]. The region is known for large-amplitude internal waves (LAIW) and the corals at Ko Racha Yai were regularly exposed to LAIW, but the short-term cooling events caused by LAIW have not been described as source of thermal stress leading to bleaching. We note that the reconstructed amplified cooling during the strong combined positive Indian Ocean Dipole (pIOD) and El Niño event in 1997/98 might be a relatively rare event in modern times, as 1997 was the most extreme pIOD event of the 20th century[8]. However, even if this was an unusual and extreme event, we do not observe any indicators, such as a stress band[9], decreased growth rate[10] (Supplementary Figs. 1, 12), or disruption in the annual cycle of coral Sr/Ca and Mg/Ca[11,12] during 1997/98. This is also true for the other pronounced cooling events identified in the coral Sr/Ca record. Therefore, we conclude that the coral Sr/Ca-based cooling events are a true signal, reflecting subsurface temperature variability induced by pronounced thermocline shoaling and LAIW in the Andaman Sea, which are not captured by satellite sea surface temperature (SST) products[13].

Supplementary Note 2

Controlling factors of the coral $\delta^{13}C$ annual cycle at Ko Racha Yai

The annual cycle of Ko Racha Yai coral skeletal $\delta^{13}C$ reveals a double-peak pattern similar to that observed in coral Sr/Ca temperature and satellite SST (Supplementary Fig. 2a,b), suggesting common seasonal forcing by the Asian monsoon. The most negative coral $\delta^{13}C$ value occurs in April, and the most positive values in July-September. A second less pronounced negative peak occurs in November (Supplementary Fig. 2b). Importantly, this coral $\delta^{13}C$ pattern cannot be solely explained by light availability for photosynthesis, which has been suggested to be one of the main controlling factors of $\delta^{13}C$ in coral skeletons. Photosynthesis by algae symbionts preferentially fixes ^{12}C into organic carbon, resulting during the calcification process in a skeleton that is relatively enriched in ^{13}C (ref. [14]). This generally leads to more positive coral skeletal $\delta^{13}C$ values during periods of high light availability and higher photosynthesis[15-20]. However, at Ko Racha Yai the coral $\delta^{13}C$ annual cycle has the most positive values during the peak of the wet southwest (SW) monsoon season (June-July-August), when solar irradiance and outgoing longwave radiation (OLR) reach their lowest values (Supplementary Fig. 2c), i.e., when light availability is the lowest. In contrast, more negative coral $\delta^{13}C$ values characterise the dry northeast (NE) monsoon season (November-April) that has less cloud cover, rainfall, runoff, i.e., more light availability.

An explanation for the coral $\delta^{13}C$ annual cycle at Ko Racha Yai (Supplementary Fig. 2b) can be partly derived from the similarity of the annual cycles of solar irradiance, OLR, and chlorophyll *a* in the region. These suggest that the higher light availability during the dry NE monsoon season (November-April) is associated with a higher overall water column productivity and phytoplankton availability compared to the SW monsoon season (May-October) (Supplementary Fig. 2c,d). Higher primary productivity can increase coral consumption on zooplankton and dissolved and particulate organic carbon (i.e., heterotrophy)[21], resulting in a more negative coral $\delta^{13}C$ signal[19,20], consistent with what is observed at Ko Racha Yai during the NE monsoon season. Zooplankton typically has more negative $\delta^{13}C$ values ranging from -14 to -25‰ or even lower[22] compared to coral skeletons. Additionally, nutrient-rich deeper waters advected by thermocline shoaling and LAIW during the NE monsoon provide additional food for the coral[23], further consistent with the more negative skeletal $\delta^{13}C$ values during this season. Conversely, the wet SW monsoon season brings more clouds and rainfall, resulting in decreased primary productivity (Supplementary Fig. 2c,d). Less oceanic productivity may reduce coral heterotrophic feeding and thus increases the relative contribution of endosymbiotic photosynthesis (i.e., autotrophy) to the skeletal $\delta^{13}C$ signal. Thus, the most positive values of coral $\delta^{13}C$ are observed during the SW monsoon despite of less light availability during this season. In summary, we interpret the seasonal variation of Ko Racha Yai coral $\delta^{13}C$ as to be mainly influenced by changes in the coral's autotrophy-heterotrophy balance throughout the year.

Supplementary Note 3

Coral $\delta^{13}C$ signal during the 1998 surface heat stress and bleaching event

An enhanced upwelling influence and subsurface cooling of reefs through thermocline shoaling and LAIW as inferred for the 1998 surface heat stress and bleaching event in the southern Andaman Sea is supported by an anomalously high chlorophyll *a* concentration around Ko Racha Yai (Supplementary Fig. 13a-d). Importantly, the effects of a higher turbidity[24] reducing light penetration into the water column and other consequences of stronger upwelling such as increased nutrient availability[23] were potentially outpaced by a higher light availability during this year. The concurrent combined pIOD and El Niño event 1997/98 resulted in an anomalously low sea surface height (SSH) and reduced cloud cover in the area[25,26] (Supplementary Fig. 13d-f), potentially increasing the availability of light for the coral, thereby supporting more coral autotrophy and thus more positive coral skeletal $\delta^{13}C$ (ref. [16]).

Shifts in light exposure can have profound impacts on coral skeletal $\delta^{13}C$. Sudden shifts in light exposure due to abrupt seismic displacements[16,27] in the equatorial eastern Indian Ocean were shown to cause step-changes in coral $\delta^{13}C$ of 0.2-0.3 ‰ m^{-1} up to 0.6 ‰ m^{-1}. Using these values as benchmark, converting the SSH anomalies of about -30 cm observed in the Andaman Sea in 1997/98 (Supplementary Fig. 13e) results in a potential coral $\delta^{13}C$ shift at Ko Racha Yai of ~0.06-0.18‰ towards positive values. Together with the effect of an anomalously low cloud cover (Supplementary Fig. 13f), this may have compensated any effects of nutrient excess or enhanced feeding from pronounced thermocline shoaling and LAIW in 1997/98, resulting in the maintenance of more positive coral $\delta^{13}C$ values.

Supplementary Table 1. Ordinary least squares (OLS) regression equations and Pearson correlation coefficients, *r* (r^2) between Ko Racha Yai coral Sr/Ca and gridded sea surface temperature (SST) products[13,28-31] for monthly and annual average (April-March) records (1985-2010) using all data, and with coral Sr/Ca-based cooling events excluded. Cooling events were identified as extreme values in monthly coral Sr/Ca records (Fig. 2a, Supplementary Fig. 5). Root mean square residuals (RMSR) calculated as the square root of the sum of all residual values.

SST product	OLS regression equation	r (r^2)	p	RMSR	n
**All data (1985-2010)**					
Monthly					
NOAA CRW	Sr/Ca = -0.043 (± 0.003) × SST + 10.065 (± 0.082)	-0.66 (0.44)	<<0.01	0.034	305
OISSTv2.1	Sr/Ca = -0.037 (± 0.003) × SST + 9.882 (± 0.091)	-0.56 (0.31)	<<0.01	0.038	305
OISSTv2	Sr/Ca = -0.037 (± 0.003) × SST + 9.896 (± 0.092)	-0.56 (0.31)	<<0.01	0.038	305
HadISST	Sr/Ca = -0.037 (± 0.004) × SST + 9.891 (± 0.112)	-0.48 (0.23)	<<0.01	0.04	305
ERSSTv5	Sr/Ca = -0.038 (± 0.004) × SST + 9.907 (± 0.105)	-0.51 (0.26)	<<0.01	0.039	305
Annual					
NOAA CRW	Sr/Ca = -0.052 (± 0.011) × SST + 10.317 (± 0.33)	-0.68 (0.46)	<<0.01	0.023	26
OISSTv2.1	Sr/Ca = -0.049 (± 0.012) × SST + 10.226 (± 0.356)	-0.63 (0.4)	<0.01	0.024	26
OISSTv2	Sr/Ca = -0.046 (± 0.014) × SST + 10.171 (± 0.411)	-0.56 (0.31)	<0.01	0.026	26
HadISST	Sr/Ca = -0.055 (± 0.019) × SST + 10.422 (± 0.542)	-0.52 (0.27)	<0.01	0.026	26
ERSSTv5	Sr/Ca = -0.056 (± 0.016) × SST + 10.452 (± 0.46)	-0.59 (0.35)	<0.01	0.025	26
**Cooling events excluded (1985-2010)**					
Monthly					
NOAA CRW	Sr/Ca = -0.038 (± 0.003) × SST + 9.918 (± 0.075)	-0.65 (0.43)	<<0.01	0.03	298
OISSTv2.1	Sr/Ca = -0.032 (± 0.003) × SST + 9.746 (± 0.082)	-0.55 (0.31)	<<0.01	0.033	298
OISSTv2	Sr/Ca = -0.032 (± 0.003) × SST + 9.751 (± 0.083)	-0.55 (0.3)	<<0.01	0.034	298
HadISST	Sr/Ca = -0.032 (± 0.003) × SST + 9.737 (± 0.101)	-0.47 (0.22)	<<0.01	0.035	298
ERSSTv5	Sr/Ca = -0.033 (± 0.003) × SST + 9.778 (± 0.093)	-0.52 (0.27)	<<0.01	0.034	298
Annual					
NOAA CRW	Sr/Ca = -0.048 (± 0.011) × SST + 10.212 (± 0.307)	-0.68 (0.47)	<<0.01	0.021	26
OISSTv2.1	Sr/Ca = -0.046 (± 0.011) × SST + 10.138 (± 0.322)	-0.64 (0.41)	<<0.01	0.022	26
OISSTv2	Sr/Ca = -0.042 (± 0.013) × SST + 10.03 (± 0.374)	-0.55 (0.31)	<0.01	0.023	26
HadISST	Sr/Ca = -0.05 (± 0.017) × SST + 10.269 (± 0.504)	-0.51 (0.26)	<0.01	0.024	26
ERSSTv5	Sr/Ca = -0.052 (± 0.015) × SST + 10.322 (± 0.423)	-0.59 (0.35)	<0.01	0.023	26

Supplementary Table 2. Same as Supplementary Table 1 but using reduced major axis (RMA) regression.

SST product	RMA regression equation	*r* (r^2)	*p*	RMSR	*n*
**All data (1985-2010)**					
Monthly					
NOAA CRW	Sr/Ca = -0.065 (± 0.003) × SST + 10.707 (± 0.09)	-0.66 (0.44)	<<0.01	0.038	305
OISSTv2.1	Sr/Ca = -0.066 (± 0.004) × SST + 10.722 (± 0.103)	-0.56 (0.31)	<<0.01	0.043	305
OISSTv2	Sr/Ca = -0.066 (± 0.004) × SST + 10.751 (± 0.105)	-0.56 (0.31)	<<0.01	0.043	305
HadISST	Sr/Ca = -0.077 (± 0.004) × SST + 11.042 (± 0.13)	-0.48 (0.23)	<<0.01	0.047	305
ERSSTv5	Sr/Ca = -0.073 (± 0.004) × SST + 10.946 (± 0.121)	-0.51 (0.26)	<<0.01	0.045	305
Annual					
NOAA CRW	Sr/Ca = -0.076 (± 0.012) × SST + 11.02 (± 0.36)	-0.68 (0.46)	<<0.01	0.025	26
OISSTv2.1	Sr/Ca = -0.077 (± 0.014) × SST + 11.056 (± 0.394)	-0.63 (0.4)	<0.01	0.027	26
OISSTv2	Sr/Ca = -0.083 (± 0.016) × SST + 11.241 (± 0.465)	-0.56 (0.31)	<0.01	0.029	26
HadISST	Sr/Ca = -0.107 (± 0.021) × SST + 11.917 (± 0.622)	-0.52 (0.27)	<0.01	0.03	26
ERSSTv5	Sr/Ca = -0.096 (± 0.018) × SST + 11.601 (± 0.517)	-0.59 (0.35)	<0.01	0.028	26
**Cooling events excluded (1985-2010)**					
Monthly					
NOAA CRW	Sr/Ca = -0.058 (± 0.003) × SST + 10.508 (± 0.082)	-0.65 (0.43)	<<0.01	0.033	298
OISSTv2.1	Sr/Ca = -0.058 (± 0.003) × SST + 10.502 (± 0.093)	-0.55 (0.31)	<<0.01	0.038	298
OISSTv2	Sr/Ca = -0.059 (± 0.003) × SST + 10.527 (± 0.095)	-0.55 (0.3)	<<0.01	0.038	298
HadISST	Sr/Ca = -0.068 (± 0.004) × SST + 10.778 (± 0.118)	-0.47 (0.22)	<<0.01	0.041	298
ERSSTv5	Sr/Ca = -0.064 (± 0.004) × SST + 10.686 (± 0.107)	-0.52 (0.27)	<<0.01	0.04	298
Annual					
NOAA CRW	Sr/Ca = -0.071 (± 0.011) × SST + 10.864 (± 0.334)	-0.68 (0.47)	<<0.01	0.022	26
OISSTv2.1	Sr/Ca = -0.071 (± 0.012) × SST + 10.872 (± 0.355)	-0.64 (0.41)	<<0.01	0.024	26
OISSTv2	Sr/Ca = -0.075 (± 0.014) × SST + 11.013 (± 0.425)	-0.55 (0.31)	<0.01	0.027	26
HadISST	Sr/Ca = -0.098 (± 0.02) × SST + 11.679 (± 0.581)	-0.51 (0.26)	<0.01	0.028	26
ERSSTv5	Sr/Ca = -0.088 (± 0.016) × SST + 11.376 (± 0.475)	-0.59 (0.35)	<0.01	0.026	26

Supplementary Table 3. Pearson correlation values between Ko Racha Yai in-situ temperature measurements[32-34] at different water depths and gridded sea surface temperature (SST) products[13,28-31] (spatial resolutions indicated) during January 2010-September 2011 (n = 21, p << 0.01).

	NOAA CRW 0.05° x 0.05°	OISSTv2.1 0.25° x 0.25°	OISSTv2 1° x 1°	HadISST 1° x 1°	ERSSTv5 2° x 2°
West (Tanzil) (2-3 m)	0.98	0.98	0.96	0.88	0.92
West (Wall) (15 m)	0.89	0.91	0.87	0.8	0.85

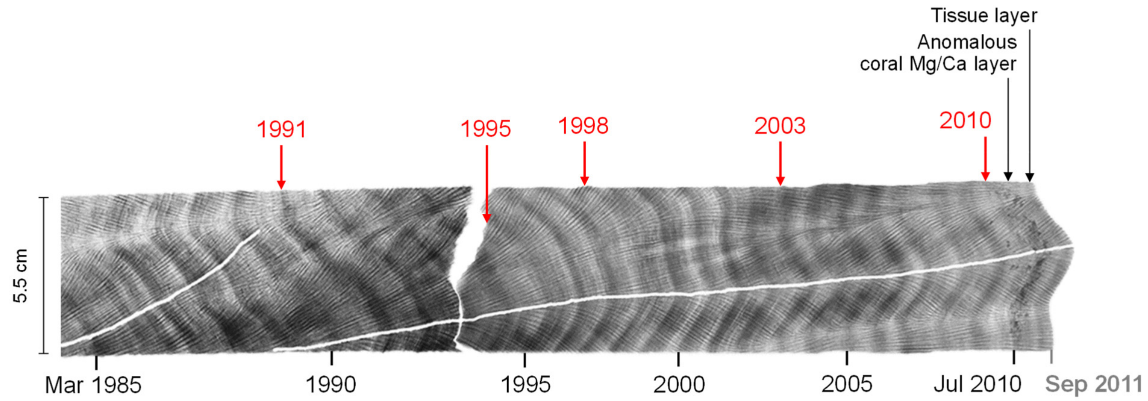

Supplementary Fig. 1. X-radiograph positive image of the top section of coral core TH3A from Siam Bay, Ko Racha Yai (Thailand), southern Andaman Sea. Coral growth is from left to right. A pair of high-density (dark colour) and low-density (bright colour) bands represents one year of coral growth. High-density bands are precipitated during the southwest monsoon season, low-density during the northeast monsoon season. White dotted lines: microsampling transect. The years of skeleton precipitation are indicated (black). The tissue layer and a layer characterised by anomalous coral Mg/Ca ratios and microboring traces are indicated, and excluded from this study. Red arrows: years with observed major bleaching events in the southern Andaman Sea[35,36].

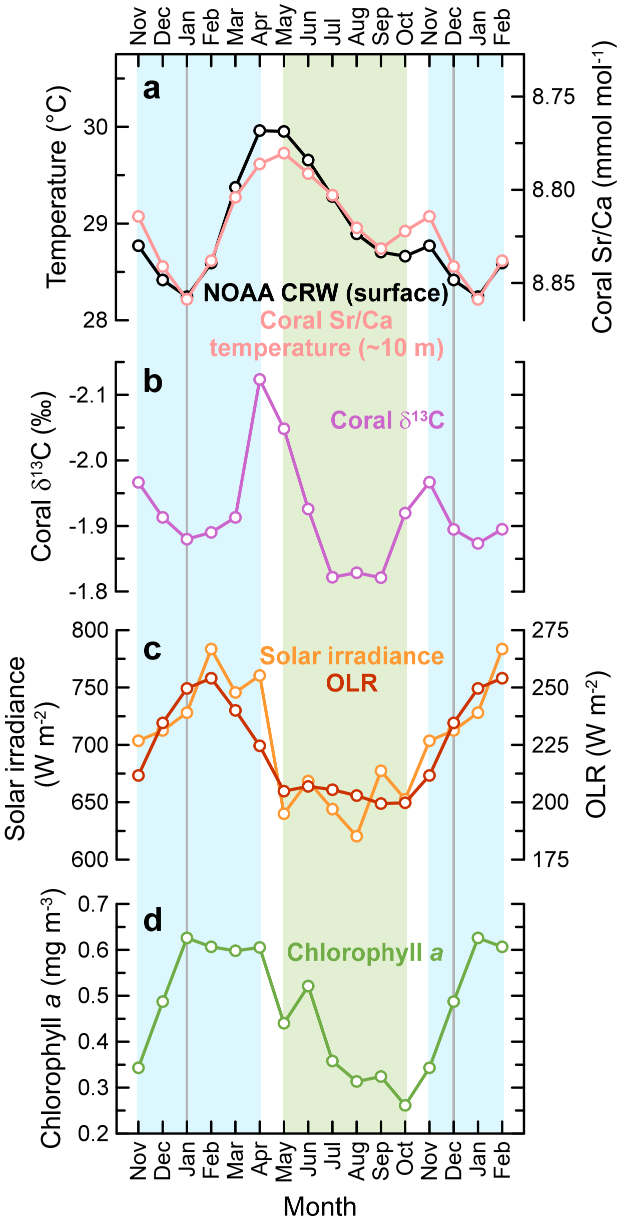

Supplementary Fig. 2. Monthly climatology of Ko Racha Yai coral skeletal geochemical and isotopic records and environmental parameters. (a) Coral Sr/Ca temperature and satellite sea surface temperature (SST) (NOAA CRW[13], centred at 7.625°N, 98.375°E) scaled with a coral Sr/Ca-temperature relationship of -0.0607 mmol mol^{-1} per °C (ref. [37]) (1985-2010). (b) Coral $\delta^{13}C$ (1985-2010). (c) Surface solar irradiance (FRESCO v6[38], centred at 7.6°N, 98.367°E, 2002-2010) and outgoing longwave radiation (OLR) (NOAA NCAR[39], centred at 7.5°N, 97.5°E, 1985-2010). (d) Surface chlorophyll *a* concentration (SeaWIFS[40], centred at 7.625°N, 98.375°E, 1997-2010). Vertical bars: southwest (green) and northeast (blue) monsoon seasons[34]. Vertical grey lines indicate the beginning and end of one annual cycle.

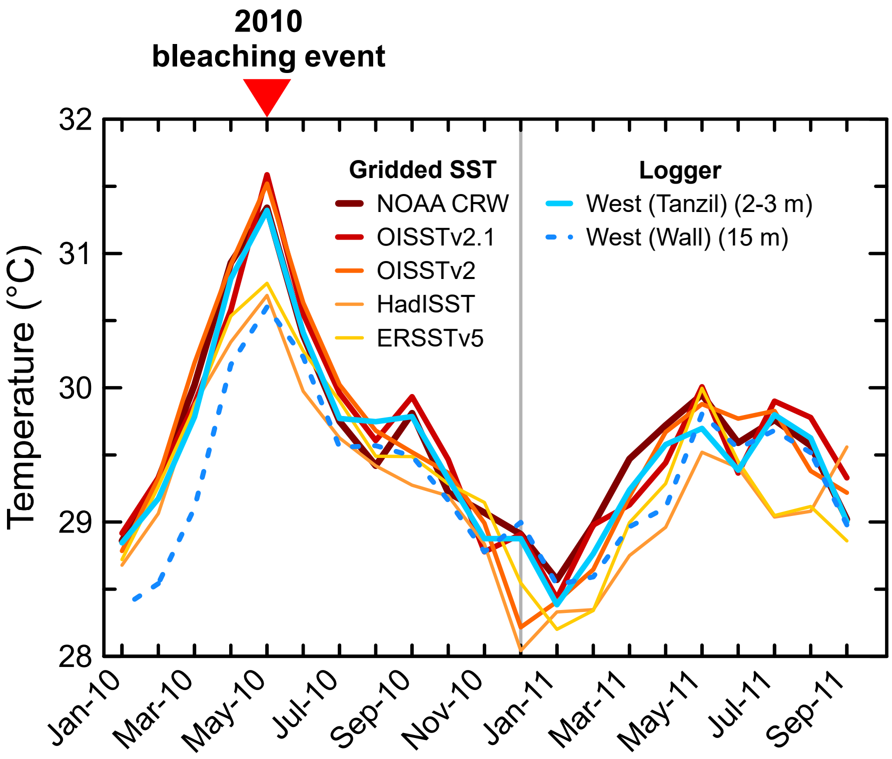

Supplementary Fig. 3. Comparison between Ko Racha Yai monthly averaged in-situ temperatures derived from logger measurements[32-34] at shallow-reef habitats (see Fig. 1 for locations) with monthly gridded sea surface temperature (SST) products[13,28-31]. Red triangle: peak of marine heatwave during the 2010 bleaching event in the southern Andaman Sea, associated with the 2009/10 El Niño. Note the colder subsurface temperature compared to the surface during the latter, as well as the similar temperatures at subsurface and surface during the subsequent 2010/11 La Niña event. Vertical line marks the end of the year 2010.

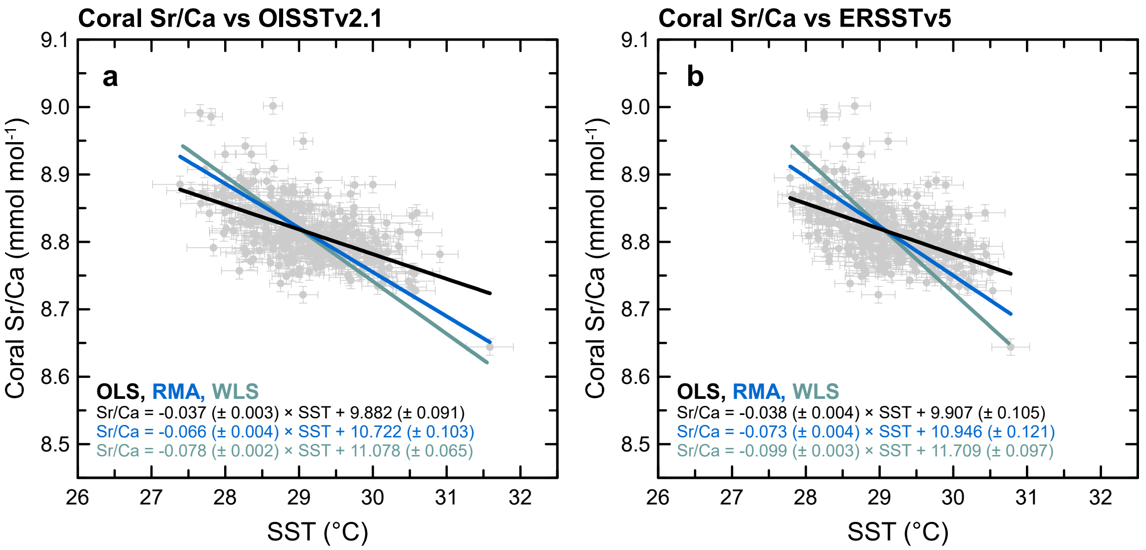

Supplementary Fig. 4. Calibration between monthly Ko Racha Yai coral Sr/Ca and sea surface temperature (SST) by using ordinary least squares (OLS), reduced major axis (RMA), and weighted linear squares (WLS) regressions. (a) With OISSTv2.1[28]. (b) With ERSSTv5[31]. WLS regression done by incorporating uncertainty (error bars, 1σ) from coral Sr/Ca (analytical error) and each SST product. WLS calculation adapted to Python following Thirumalai et al. (2011)[41].

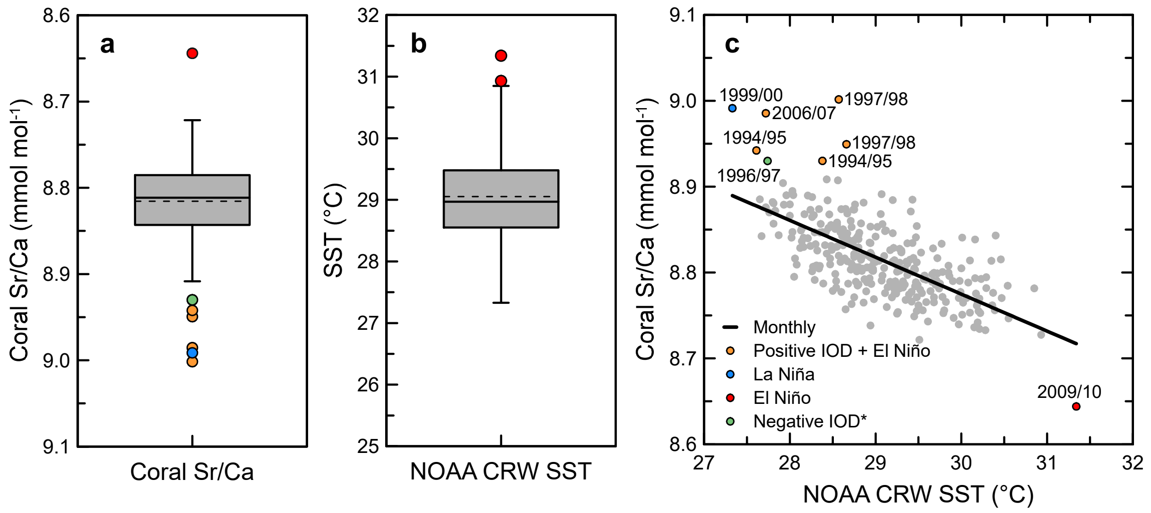

Supplementary Fig. 5. Extreme values (coloured data points) in monthly Ko Racha Yai (a) coral Sr/Ca and (b) NOAA CRW sea surface temperature[13] (SST) (1985-2010). Dashed horizontal line: mean, solid horizontal line: median. (c) Scatter plot of monthly coral Sr/Ca and NOAA CRW SST. Solid line: regression line. Extreme values and associated climate events are indicated by the colour of data points (a, b, c), and corresponding years are indicated (c). Mark * on the 1996/97 negative Indian Ocean Dipole (IOD) event indicates the year is not identified as interannual climate event after detrending and removing variability >7 years, following Abram et al. (2020)[8].

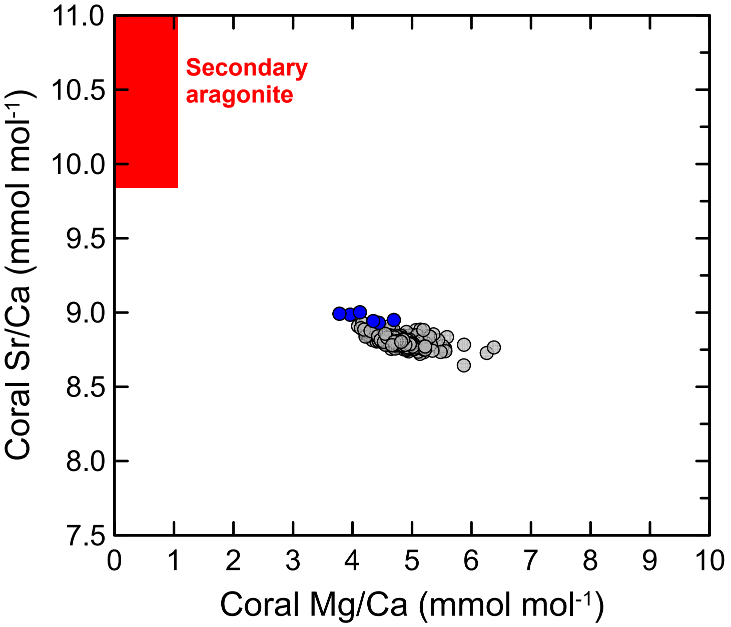

Supplementary Fig. 6. Comparison of Ko Racha Yai coral Sr/Ca and Mg/Ca values (grey, blue) with the diagenetic aragonite field (red area) defined using data from refs. [42,43] adapted from ref. [44]. Importantly, none of the coral Sr/Ca and Mg/Ca values during the coral Sr/Ca-based cooling events (blue) (Fig. 2a) encounter the diagenetic aragonite field, ruling out any influence of early diagenesis such as secondary aragonite precipitation on the coral geochemical proxy records.

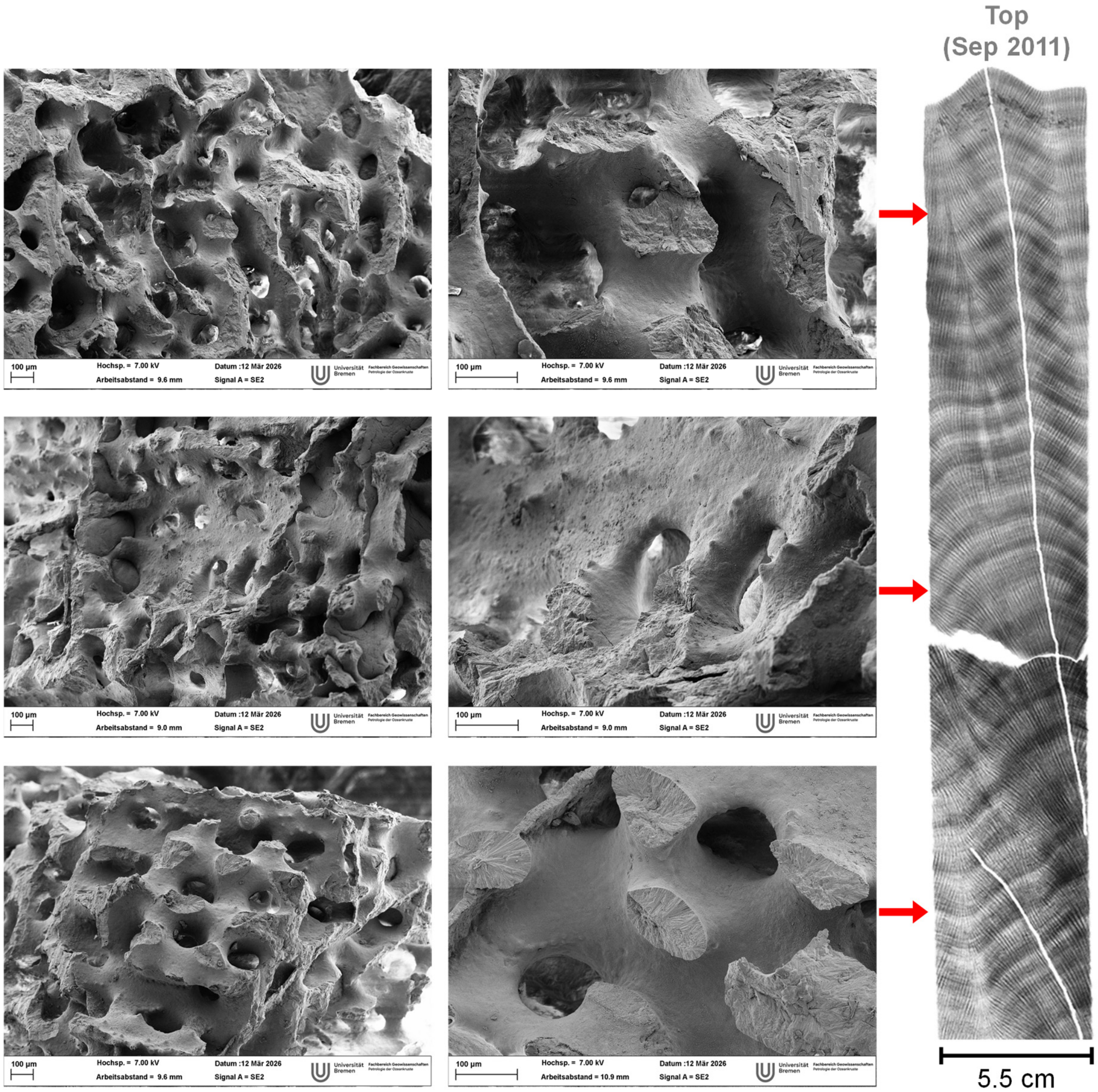

Supplementary Fig. 7. Scanning electron microscope (SEM) images taken along the microsampling transect as indicated on an X-radiograph positive image of the Ko Racha Yai coral core (TH3A). Red arrows mark where subsamples were taken for SEM analysis. No secondary aragonite or calcite cements are observed in the skeletal pore spaces, resulting in excellent preservation of primary porosity.

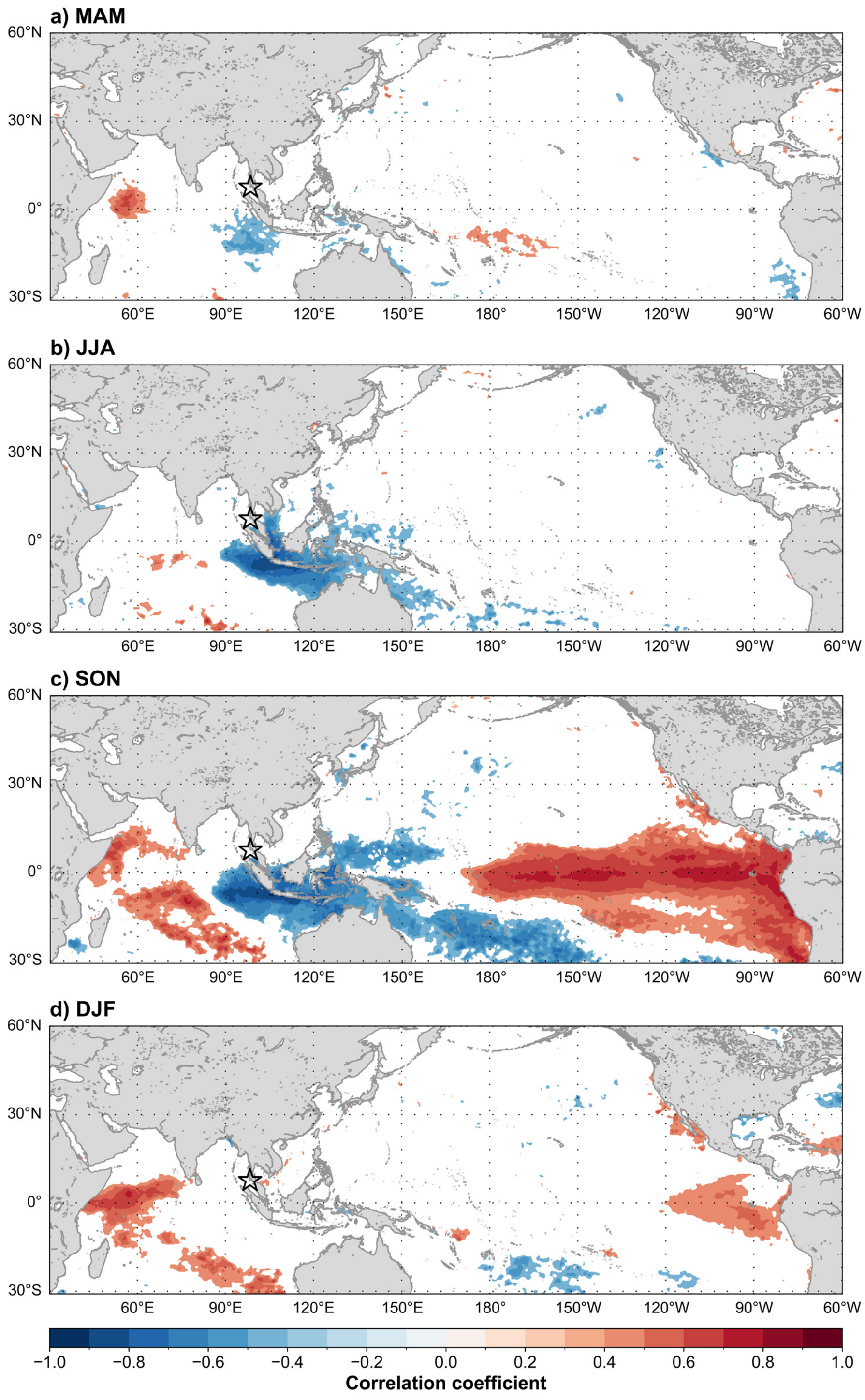

Supplementary Fig. 8. Spatial correlation maps for the monthly Dipole Mode Index (DMI) and global sea surface temperature (SST) (OISSTv2.1[28]) (1985-2010, $p < 0.05$). (a) March-April-May (MAM). (b) June-July-August (JJA). (c) September-October-November (SON). (d) December-January-February (DJF). Correlation maps calculated with KNMI Climate Explorer (https://climexp.knmi.nl/) and redrawn with Python. Datasets were detrended prior the analysis. DMI calculated with ERSSTv5[31]. Stars: Ko Racha Yai coral site.

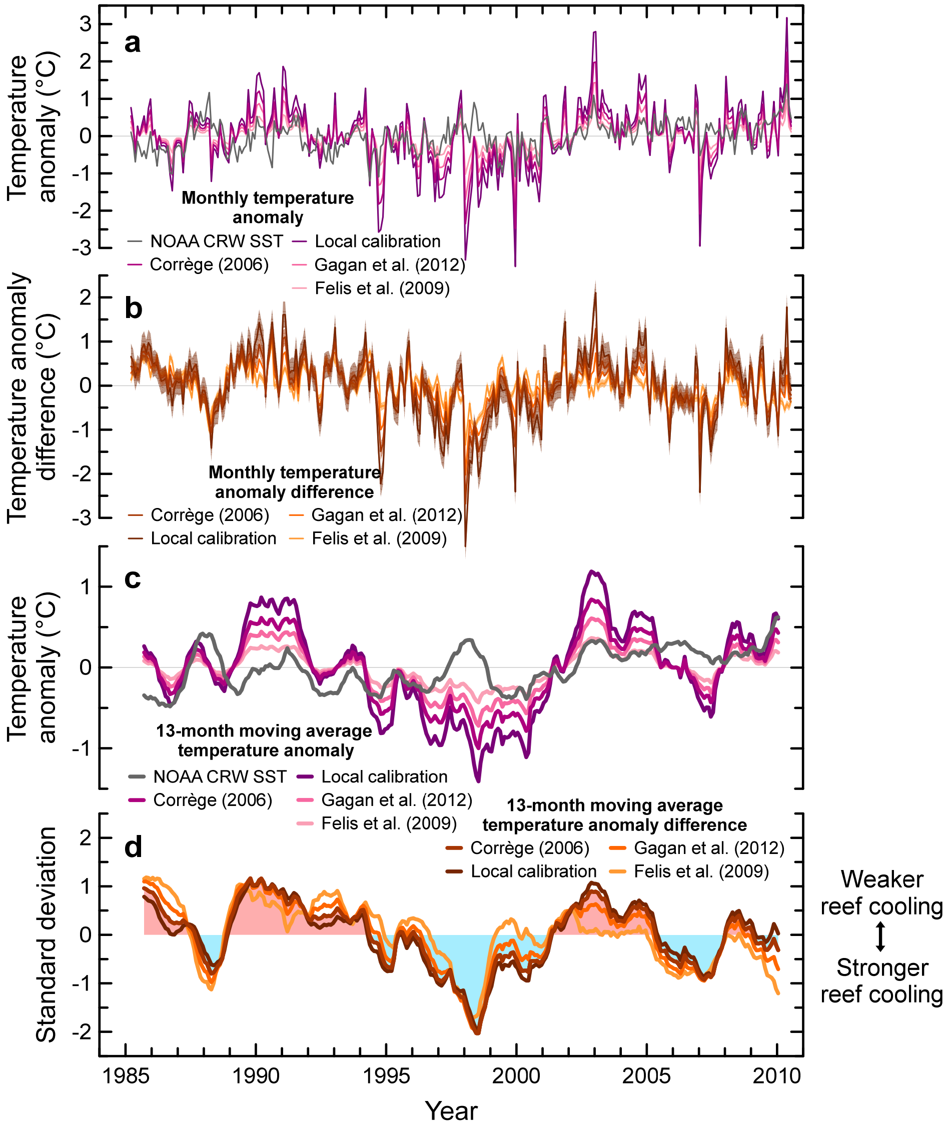

Supplementary Fig. 9. Temperature difference between surface and coral depth (~10 m). (a) Monthly anomalies of Ko Racha Yai coral Sr/Ca temperature and satellite sea surface temperature (SST) (NOAA CRW[13]). Coral Sr/Ca temperature anomaly calculated using relationships of -0.0607 mmol mol^{-1} per °C (ref. [37]), -0.043 mmol mol^{-1} per °C (local calibration, see Table 1 and Supplementary Table 1), -0.084 mmol mol^{-1} per °C (ref. [45]), and -0.14 mmol mol^{-1} per °C (ref. [46]). (b) Monthly temperature anomaly difference calculated by subtracting anomalies of NOAA CRW SST from coral Sr/Ca temperature in (a). Shadings represent uncertainty (1σ) calculated from coral Sr/Ca (analytical error) and the error of each coral Sr/Ca-temperature relationship. (c) Same as (a) but for 13-month moving averages. (d) 13-month moving average of standardised values in (b).

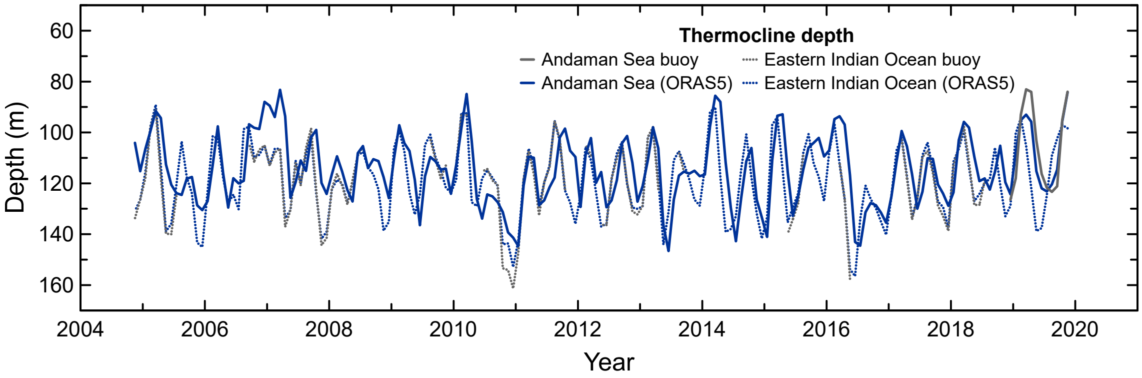

Supplementary Fig. 10. Comparison of monthly thermocline depth in the Andaman Sea and the equatorial eastern Indian Ocean between reanalysis data and available buoy measurements. Thermocline depth taken as depth of 20°C isotherm[47]. Reanalysis data (ORAS5[48]) centred at buoy locations in the central Andaman Sea (9.6°N, 95.6°E)[49,50] and equatorial eastern Indian Ocean (0°, 90°E)[51].

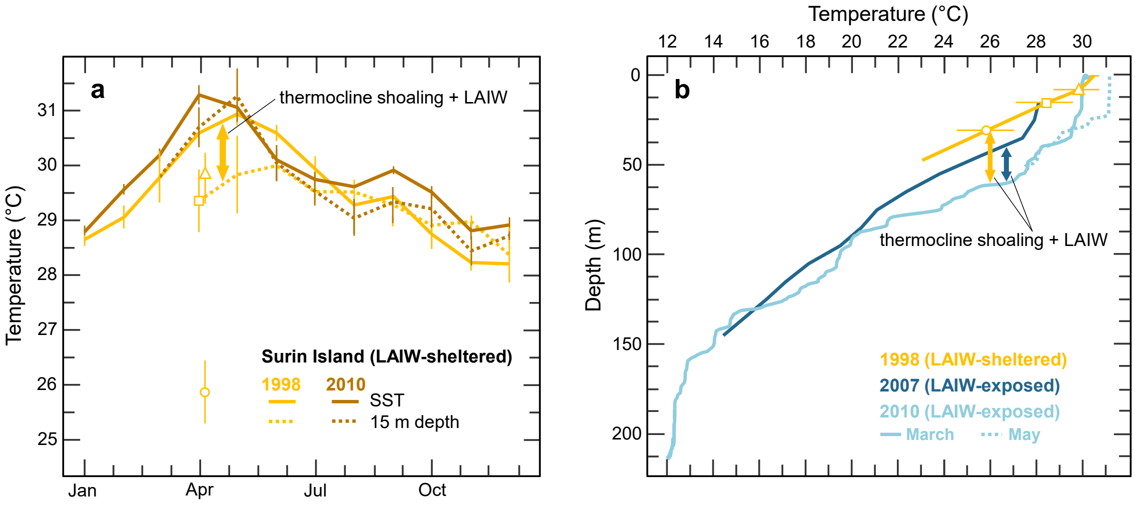

Supplementary Fig. 11. (a) Monthly averaged NOAA CRW sea surface temperature (SST)[13] (solid lines) and in-situ temperature at 15 m depth (dashed lines) in 1998 (ref. [35]) and 2010 (ref. [34]) from Surin Island (LAIW-sheltered side). (b) Temperature versus depth profiles for 1998, 2007, and 2010. Temperatures were measured in April 1998 at 10 m (triangle), 15 m (square), and 30 m (circle) depth at Surin Island (LAIW-sheltered side), in 2007 by a temperature logger mooring at Miang Island (LAIW-exposed side), and in 2010 by CTD casts at Surin Island (LAIW-exposed side) during March (solid line) and May (dashed line)[33]. Error bars: standard deviation values. Arrows highlight subsurface reef cooling by thermocline shoaling and LAIW. See Fig. 1 for locations of temperature measurements.

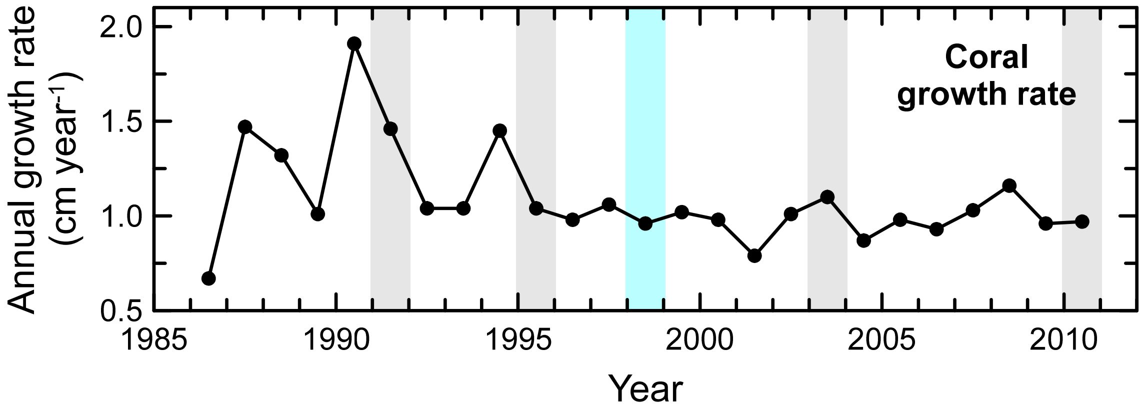

Supplementary Fig. 12. Ko Racha Yai annual coral growth rate. Years with observed major southern Andaman Sea bleaching events are indicated (vertical bars)[35,36]. The 1998 event is highlighted (blue). Annual growth rate was calculated from the distance between the coral Sr/Ca maximum in a given year to the coral Sr/Ca maximum of the following year, i.e., from winter (~January) to the following winter.

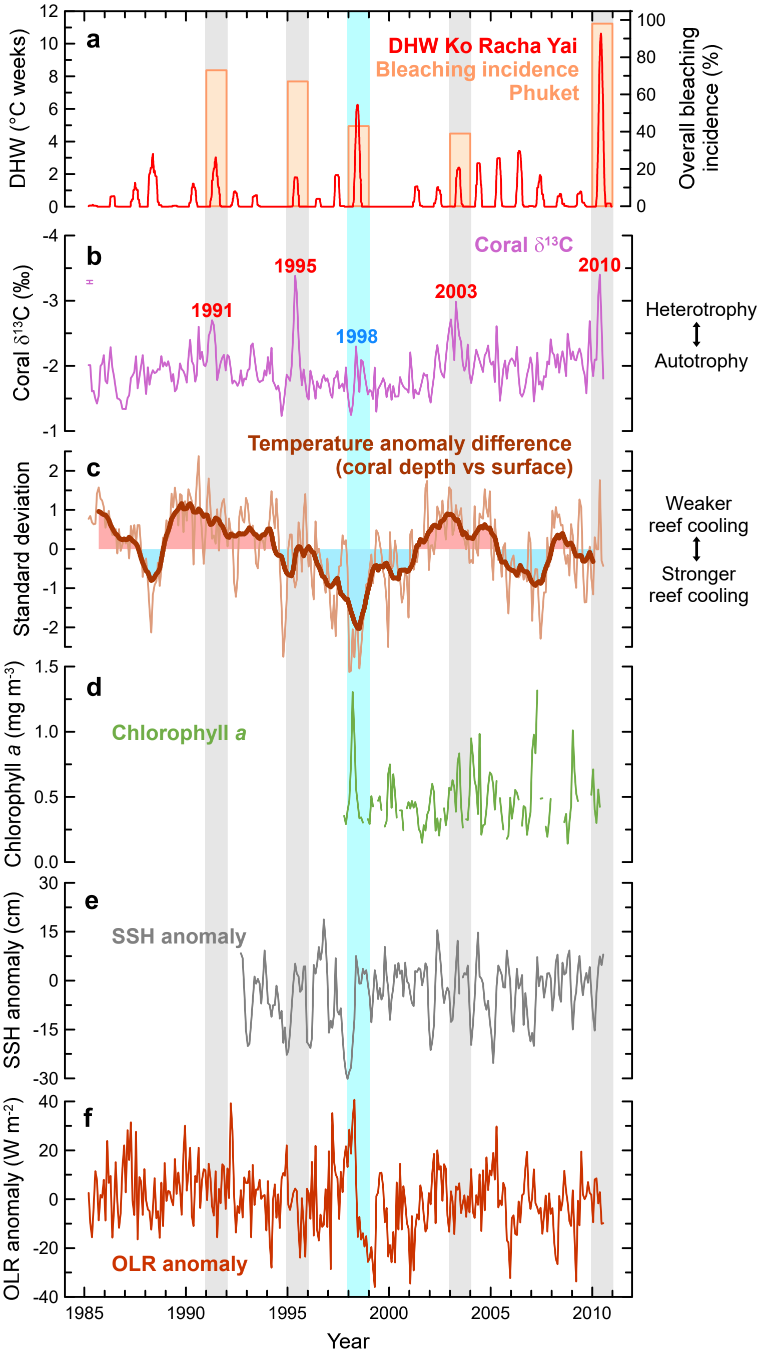

Supplementary Fig. 13. Coral stress response and environmental changes during major southern Andaman Sea bleaching events. (a-c) Same as Fig. 6. (d) Surface chlorophyll *a* concentration[40] (see Supplementary Fig. 2d for details). (e) Sea surface height (SSH) anomaly from altimetry and tide gauge at Phuket (see Fig. 4d for details). (f) Outgoing longwave radiation (OLR)[39] anomaly (see Supplementary Fig. 2c for details). More positive OLR values represent less cloud cover and high light availability, more negative OLR values indicate more cloud cover and less light availability. Monthly values (d-f). Anomalies relative to climatology of the period covered by each dataset. Years with observed major southern Andaman Sea bleaching events are indicated (vertical bars)[35,36]. The 1998 event is highlighted (blue).

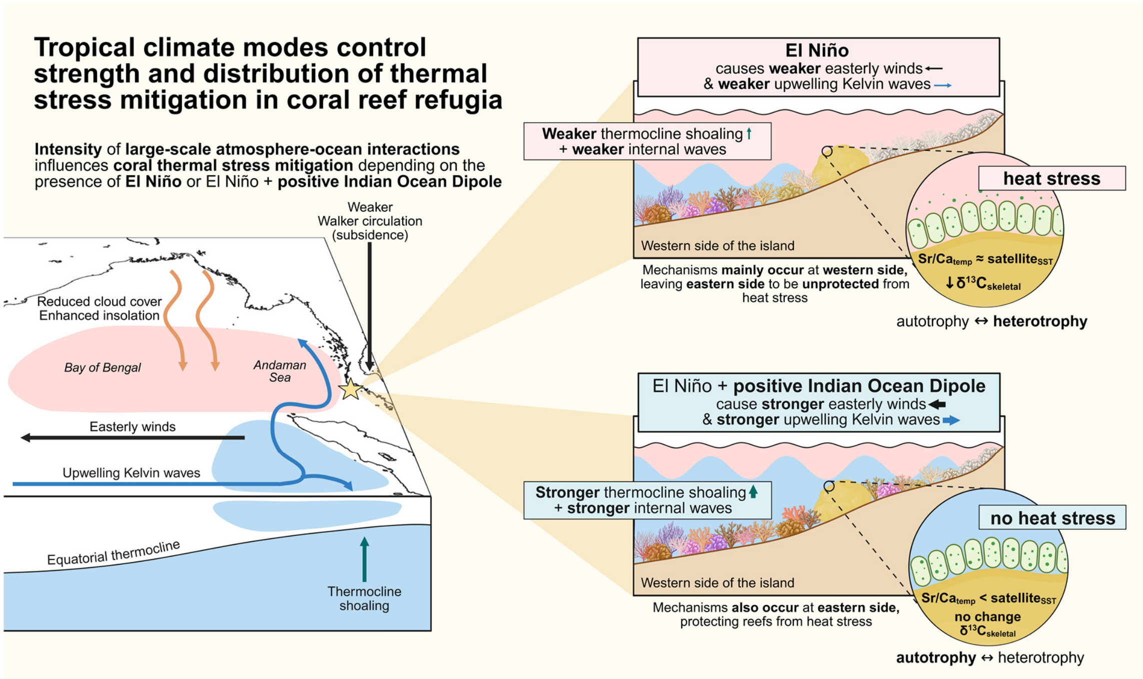

Supplementary Fig. 14. Schematic diagram illustrating how tropical climate modes control coral thermal stress mitigation by thermocline shoaling and large-amplitude internal waves in the southern Andaman Sea via remote forcing from the equatorial eastern Indian Ocean. Created in BioRender. Camelia, H. (2026) https://BioRender.com/kkpolql.

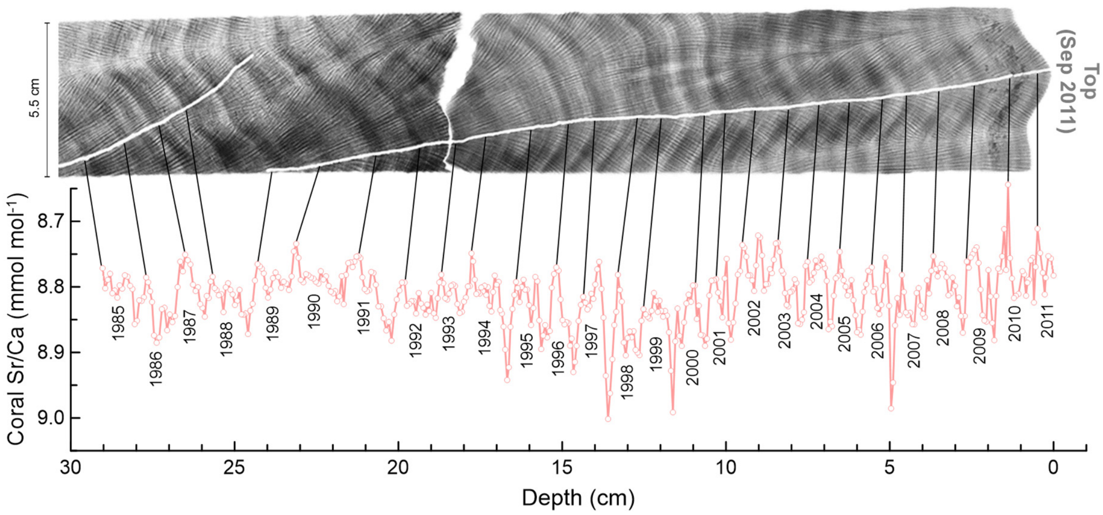

Supplementary Fig. 15. (top) X-radiograph positive image of the top section of coral core TH3A from Siam Bay, Ko Racha Yai (Thailand), southern Andaman Sea (same as Supplementary Fig. 1). A pair of high-density (dark colour) and low-density (bright colour) bands represents one year of coral growth. High-density bands are precipitated during the southwest monsoon season, low-density during the northeast monsoon season. White dotted lines: microsampling transect. (bottom) Measured coral Sr/Ca and the inferred year of coral growth/skeleton precipitation. Black lines indicate the position on the X-radiograph for coral Sr/Ca raw data that correspond to annual maxima in the NOAA CRW SST[13] for Ko Racha Yai.

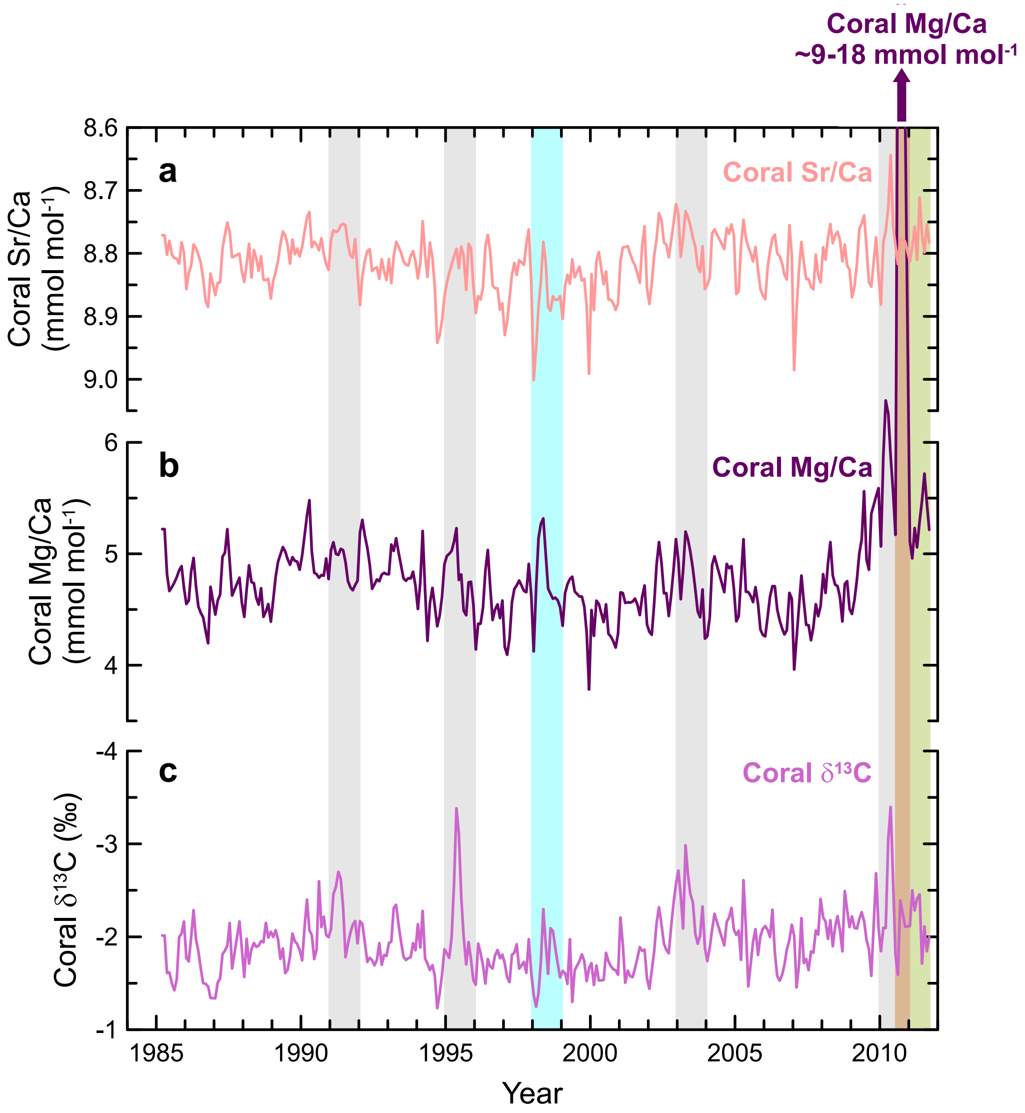

Supplementary Fig. 16. Ko Racha Yai monthly coral skeletal records from March 1985 to September 2011 (top of core). The graphs depict (a) coral Sr/Ca temperature, (b) coral Mg/Ca, and (c) coral $\delta^{13}C$ records. Tissue layer (green) and layer with anomalously high coral Mg/Ca and microboring traces (brown) excluded from reconstructions. Years with observed major southern Andaman Sea bleaching events are indicated (vertical bars)[35,36]. The 1998 event is highlighted (blue). All geochemical and isotopic records measured from the same sample powder.

References

1 Alibert, C. & McCulloch, M. T. Strontium/calcium ratios in modern *Porites* corals from the Great Barrier Reef as a proxy for sea surface temperature: Calibration of the thermometer and monitoring of ENSO. *Paleoceanography* **12**, 345-363. https://doi.org/10.1029/97PA00318 (1997).

2 Cohen, A. L. & Hart, S. R. The effect of colony topography on climate signals in coral skeleton. *Geochim. Cosmochim. Acta* **61**, 3905-3912. https://doi.org/10.1016/S0016-7037(97)00200-7 (1997).

3 DeLong, K. L., Quinn, T. M., Taylor, F. W., Shen, C.-C. & Lin, K. Improving coral-base paleoclimate reconstructions by replicating 350 years of coral Sr/Ca variations. *Palaeogeogr. Palaeoclimatol. Palaeoecol.* **373**, 6-24. https://doi.org/10.1016/j.palaeo.2012.08.019 (2013).

4 Müller, A., Gagan, M. K. & McCulloch, M. T. Early marine diagenesis in corals and geochemical consequences for paleoceanographic reconstructions. *Geophys. Res. Lett.* **28**, 4471-4474. https://doi.org/10.1029/2001GL013577 (2001).

5 Quinn, T. M. & Taylor, F. W. SST artifacts in coral proxy records produced by early marine diagenesis in a modern coral from Rabaul, Papua New Guinea. *Geophys. Res. Lett.* **33**, L04601. https://doi.org/10.1029/2005GL024972 (2006).

6 Hendy, E. J., Gagan, M. K., Lough, J. M., McCulloch, M. & deMenocal, P. B. Impact of skeletal dissolution and secondary aragonite on trace element and isotopic climate proxies in *Porites* corals. *Paleoceanography* **22**, PA4101. https://doi.org/10.1029/2007PA001462 (2007).

7 Rich, W. A., Carvalho, S. & Berumen, M. L. Coral bleaching due to cold stress on a central Red Sea reef flat. *Ecol. Evol.* **12**, e9450. https://doi.org/10.1002/ece3.9450 (2022).

8 Abram, N. J. et al. Coupling of Indo-Pacific climate variability over the last millennium. *Nature* **579**, 385-392. https://doi.org/10.1038/s41586-020-2084-4 (2020).

9 Cantin, N. E. & Lough, J. M. Surviving coral bleaching events: *Porites* growth anomalies on the Great Barrier Reef. *PLoS ONE* **9**, e88720. https://doi.org/10.1371/journal.pone.0088720 (2014).

10 Hetzinger, S., Pfeiffer, M., Dullo, W. C., Zinke, J. & Garbe-Schönberg, D. A change in coral extension rates and stable isotopes after El Niño-induced coral bleaching and regional stress events. *Sci. Rep.* **6**, 32879. https://doi.org/10.1038/srep32879 (2016).

11 D'Olivo, J. P. et al. Long-term impacts of the 1997-1998 bleaching event on the growth and resilience of massive *Porites* corals from the Central Red Sea. *Geochem. Geophys. Geosys.* **20**, 2936-2954. https://doi.org/10.1029/2019GC008312 (2019).

12 D'Olivo, J. P. & McCulloch, M. T. Response of coral calcification and calcifying fluid composition to thermally induced bleaching stress. *Sci. Rep.* **7**, 2207. https://doi.org/10.1038/s41598-017-02306-x (2017).

13 Skirving, W. et al. CoralTemp and the Coral Reef Watch Coral Bleaching Heat Stress Product Suite Version 3.1. *Remote Sens.* **12**, 3856. https://doi.org/10.3390/rs12233856 (2020).

14 Swart, P. K. Carbon and oxygen isotope fractionation in scleractinian corals: a review. *Earth-Sci. Rev.* **19**, 51-80. https://doi.org/10.1016/0012-8252(83)90076-4 (1983).

15 Swart, P. K., Leder, J. J., Szmant, A. M. & Dodge, R. E. The origin of variations in the isotopic record of scleractinian corals: II. Carbon. *Geochim. Cosmochim. Acta* **60**, 2871-2885. https://doi.org/10.1016/0016-7037(96)00119-6 (1996).

16 Gagan, M. K. et al. Coral $^{13}C/^{12}C$ records of vertical seafloor displacement during megathrust earthquakes west of Sumatra. *Earth Planet. Sci. Lett.* **432**, 461-471. https://doi.org/10.1016/j.epsl.2015.10.002 (2015).

17 Linsley, B. K. et al. Coral carbon isotope sensitivity to growth rate and water depth with paleo-sea level implications. *Nat. Commun.* **10**, 2056. https://doi.org/10.1038/s41467-019-10054-x (2019).

18 Felis, T., Hinestrosa, G., Köhler, P. & Webster, J. Role of the deglacial buildup of the Great Barrier Reef for the global carbon cycle. *Geophys. Res. Lett.* **49**, e2021GL096495. https://doi.org/10.1029/2021GL096495 (2022).

19 Felis, T., Pätzold, J., Loya, Y. & Wefer, G. Vertical water mass mixing and plankton blooms recorded in skeletal stable carbon isotopes of a Red Sea coral. *J. Geophys. Res. Oceans* **103**, 30731-30739. https://doi.org/10.1029/98JC02711 (1998).

20 Grottoli, A. G. & Wellington, G. M. Effect of light and zooplankton on skeletal $\delta^{13}C$ values in the eastern Pacific corals *Pavona clavus* and *Pavona gigantea*. *Coral Reefs* **18**, 29-41. https://doi.org/10.1007/s003380050150 (1999).

21 Fox, M. D. et al. Gradients in primary production predict trophic strategies of mixotrophic corals across spatial scales. *Curr. Biol.* **28**, 3355-3363.e3354. https://doi.org/10.1016/j.cub.2018.08.057 (2018).

22 Rau, G. H., Teyssie, J. L., Rassoulzadegan, F. & Fowler, S. W. $^{13}C/^{12}C$ and $^{15}N/^{14}N$ variations among size-fractionated marine particles: implications for their origin and trophic relationships. *Mar. Ecol. Prog. Ser.* **59**, 33-38. https://doi.org/10.3354/meps059033 (1990).

23 Roder, C. et al. Trophic response of corals to large amplitude internal waves. *Mar. Ecol. Prog. Ser.* **412**, 113-128. https://doi.org/10.3354/meps08707 (2010).

24 Schmidt, G. M. et al. Coral community composition and reef development at the Similan Islands, Andaman Sea, in response to strong environmental variations. *Mar. Ecol. Prog. Ser.* **456**, 113-126. https://doi.org/10.3354/meps09682 (2012).

25 Webster, P. J., Moore, A. M., Loschnigg, J. P. & Leben, R. R. Coupled ocean–atmosphere dynamics in the Indian Ocean during 1997–98. *Nature* **401**, 356-360. https://doi.org/10.1038/43848 (1999).

26 Ummenhofer, C. C., D'Arrigo, R. D., Anchukaitis, K. J., Buckley, B. M. & Cook, E. R. Links between Indo-Pacific climate variability and drought in the Monsoon Asia Drought Atlas. *Clim. Dyn.* **40**, 1319-1334. https://doi.org/10.1007/s00382-012-1458-1 (2013).

27 Ito, S., Yamazaki, A., Nishimura, Y., Yulianto, E. & Watanabe, T. Coral geochemical signals and growth responses to coseismic uplift during the great Sumatran megathrust earthquakes of 2004 and 2005. *Geochim. Cosmochim. Acta* **273**, 257-274. https://doi.org/10.1016/j.gca.2020.01.037 (2020).

28 Huang, B. et al. Improvements of the Daily Optimum Interpolation Sea Surface Temperature (DOISST) Version 2.1. *J. Clim.* **34**, 2923-2939. https://doi.org/10.1175/JCLI-D-20-0166.1 (2021).

29 Reynolds, R. W., Rayner, N. A., Smith, T. M., Stokes, D. C. & Wang, W. An Improved In Situ and Satellite SST Analysis for Climate. *J. Clim.* **15**, 1609-1625. https://doi.org/10.1175/1520-0442(2002)015%3C1609:AIISAS%3E2.0.CO;2 (2002).

30 Rayner, N. A. et al. Global analyses of sea surface temperature, sea ice, and night marine air temperature since the late nineteenth century. *J. Geophys. Res. Atmos.* **108**, 4407. https://doi.org/10.1029/2002JD002670 (2003).

31 Huang, B. et al. Extended Reconstructed Sea Surface Temperature, version 5 (ERSSTv5): Upgrades, validations, and intercomparisons. *J. Clim.* **30**, 8179-8205. https://doi.org/10.1175/JCLI-D-16-0836.1 (2017).

32 Tanzil, J. T. I. et al. Regional decline in growth rates of massive *Porites* corals in Southeast Asia. *Glob. Change Biol.* **19**, 3011-3023. https://doi.org/10.1111/gcb.12279 (2013).

33 Wall, M., Schmidt, G. M., Janjang, P., Khokiattiwong, S. & Richter, C. Differential impact of monsoon and large amplitude internal waves on coral reef development in the Andaman Sea. *PLoS ONE* **7**, e50207. https://doi.org/10.1371/journal.pone.0050207 (2012).

34 Wall, M. et al. Large-amplitude internal waves benefit corals during thermal stress. *Proc. R. Soc. B Biol. Sci.* **282**, 20140650. https://doi.org/10.1098/rspb.2014.0650 (2015).

35 Phongsuwan, N. & Chansang, H. Repeated coral bleaching in the Andaman Sea, Thailand, during the last two decades. *Phuket Mar. Biol. Cent. Res. Bull.* **71**, 19-41. (2012).

36 Brown, B. E. et al. Long-term impacts of rising sea temperature and sea level on shallow water coral communities over a ~40 year period. *Sci. Rep.* **9**, 8826. https://doi.org/10.1038/s41598-019-45188-x (2019).

37 Corrège, T. Sea surface temperature and salinity reconstruction from coral geochemical tracers. *Palaeogeogr. Palaeoclimatol. Palaeoecol.* **232**, 408-428. https://doi.org/10.1016/j.palaeo.2005.10.014 (2006).

38 Wang, P., Stammes, P., van der A, R., Pinardi, G. & van Roozendael, M. FRESCO+: an improved O_2 A-band cloud retrieval algorithm for tropospheric trace gas retrievals. *Atmos. Chem. Phys.* **8**, 6565-6576. https://doi.org/10.5194/acp-8-6565-2008 (2008).

39 Liebmann, B. & Smith, C. A. Description of a complete (interpolated) outgoing longwave radiation dataset. *Bull. Am. Met. Soc.* **77**, 1275-1277. https://www.jstor.org/stable/26233278 (1996).

40 Hu, C., Lee, Z. & Franz, B. Chlorophyll *a* algorithms for oligotrophic oceans: A novel approach based on three-band reflectance difference. *J. Geophys. Res. Oceans* **117**, C01011. https://doi.org/10.1029/2011JC007395 (2012).

41 Thirumalai, K., Singh, A. & Ramesh, R. A MATLAB™ code to perform weighted linear regression with (correlated or uncorrelated) errors in bivariate data. *J. Geol. Soc. India* **77**, 377-380. https://doi.org/10.1007/s12594-011-0044-1 (2011).

42 Allison, N. et al. Reconstruction of deglacial sea surface temperatures in the tropical Pacific from selective analysis of a fossil coral. *Geophys. Res. Lett.* **32**, L17609. https://doi.org/10.1029/2005GL023183 (2005).

43 Allison, N., Finch, A. A., Webster, J. M. & Clague, D. A. Palaeoenvironmental records from fossil corals: The effects of submarine diagenesis on temperature and climate estimates. *Geochim. Cosmochim. Acta* **71**, 4693-4703. https://doi.org/10.1016/j.gca.2007.07.026 (2007).

44 Felis, T. et al. Pronounced interannual variability in tropical South Pacific temperatures during Heinrich Stadial 1. *Nat. Commun.* **3**, 965. https://doi.org/10.1038/ncomms1973 (2012).

45 Gagan, M. K., Dunbar, G. B. & Suzuki, A. The effect of skeletal mass accumulation in *Porites* on coral Sr/Ca and $\delta^{18}O$ paleothermometry. *Paleoceanography* **27**, PA1203. https://doi.org/10.1029/2011PA002215 (2012).

46 Felis, T. et al. Subtropical coral reveals abrupt early-twentieth-century freshening in the western North Pacific Ocean. *Geology* **37**, 527-530. https://doi.org/10.1130/G25581A.1 (2009).

47 Yang, Y. et al. Kelvin waves from the equatorial Indian Ocean modulate the nonlinear internal waves in the Andaman Sea. *Environ. Res. Lett.* **18**, 094037. 10.1088/1748-9326/acf05d (2023).

48 Zuo, H., Balmaseda, M. A., Tietsche, S., Mogensen, K. & Mayer, M. The ECMWF operational ensemble reanalysis–analysis system for ocean and sea ice: a description of the system and assessment. *Ocean Sci.* **15**, 779-808. https://doi.org/10.5194/os-15-779-2019 (2019).

49 Liu, Y. Monsoon Onset Monitoring and Its Social and Ecosystem Impact data sets. *Open Sci. Framew.* https://doi.org/10.17605/OSF.IO/NZQ2C (2023).

50 Liu, Y. et al. Observed extreme freshening in the Central Andaman Sea induced by strong positive Indian Ocean Dipole. *J. Geophys. Res. Oceans* **129**, e2023JC020406. https://doi.org/10.1029/2023JC020406 (2024).

51 McPhaden, M. J. et al. RAMA: The Research Moored Array for African–Asian–Australian Monsoon Analysis and Prediction*. *Bull. Am. Meteorol. Soc.* **90**, 459-480. https://doi.org/10.1175/2008BAMS2608.1 (2009).
